# Generic residue numbering of the GAIN domain of adhesion GPCRs

**DOI:** 10.1038/s41467-024-55466-6

**Published:** 2025-01-02

**Authors:** Florian Seufert, Guillermo Pérez-Hernández, Gáspár Pándy-Szekeres, Ramon Guixà-González, Tobias Langenhan, David E. Gloriam, Peter W. Hildebrand

**Affiliations:** 1 Institute for Medical Physics and Biophysics, Leipzig University, Medical Faculty, Leipzig, Germany; 2 Institute for Medical Physics and Biophysics, Charité – Universitätsmedizin Berlin, corporate member of Freie Universität Berlin and Humboldt-Universität zu Berlin, Berlin, Germany; 3 Department of Drug Design and Pharmacology, University of Copenhagen, Universitetsparken 2, Copenhagen, Denmark; 4 Medicinal Chemistry Research Group, HUN-REN Research Center for Natural Sciences, Magyar Tudósok körútja 2., Budapest, Hungary; 5 Rudolf Schönheimer Institute of Biochemistry, Division of General Biochemistry, Medical Faculty, Leipzig University, Leipzig, Germany; 6 Comprehensive Cancer Center Central Germany (CCCG), Leipzig, Germany; 7 Institute of Biology, Faculty of Life Sciences, Leipzig University, Leipzig, Germany; 8 Center for Scalable Data Analytics and Artificial Intelligence (ScaDS.AI), Leipzig, Germany; 9 Present Address: Department of Biological Chemistry, Institute for Advanced Chemistry of Catalonia (IQAC-CSIC), Barcelona, Spain

**Keywords:** Classification and taxonomy, Protein databases, Polycystic kidney disease, G protein-coupled receptors

## Abstract

The GPCR autoproteolysis inducing (GAIN) domain is an ancient protein fold ubiquitous in adhesion G protein-coupled receptors (aGPCR). It contains a tethered agonist necessary and sufficient for receptor activation. The GAIN domain is a hotspot for pathological mutations. However, the low primary sequence conservation of GAIN domains has thus far hindered the knowledge transfer across different GAIN domains in human receptors as well as species orthologs. Here, we present a scheme for generic residue numbering of GAIN domains, based on structural alignments of over 14,000 modeled GAIN domain structures. This scheme is implemented in the GPCR database (GPCRdb) and elucidates the domain topology across different aGPCRs and their homologs in a large panel of species. We identify conservation hotspots and statistically cancer-enriched positions in human aGPCRs and show the transferability of positional and structural information between GAIN domain homologs. The GAIN-GRN scheme provides a robust strategy to allocate structural homologies at the primary and secondary levels also to GAIN domains of polycystic kidney disease 1/PKD1-like proteins, which now renders positions in both GAIN domain types comparable to one another. In summary, our work enables researchers to generate hypothesis and rationalize experiments related to GAIN domain function and pathology.

## Abstract (web summary)

Transferring information on the GAIN domain is hampered by low sequence conservation. Here, authors introduce a generic residue numbering scheme for GAIN domains to facilitate information integration as demonstrated on cancer data.

## Introduction

**Fig. 1:**
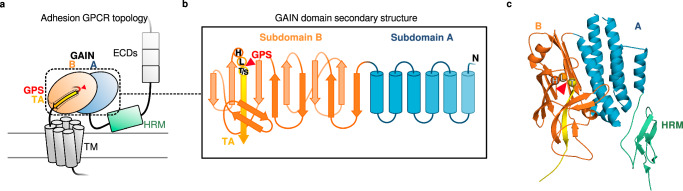
The variable topology of the G protein-coupled receptor (GPCR) autoproteolysis inducing (GAIN) domain. **a** adhesion GPCR topology with the N-terminal extracellular region composed of various extracellular domains (ECDs) and the GAIN domain directly N-terminal of the seven-transmembrane domain (TM). The GAIN domain is comprised of subdomains A and B, with the tethered agonist (TA) as the most C-terminal β-strand. The GAIN domain is frequently preceded by a hormone receptor motif (HRM) domain of unknown function. **b** the GAIN domain is composed of two subdomains, with subdomain A (blue) comprised of 2–6 α-helices with conservation decreasing toward the N-terminal boundary. The β-sandwich subdomain B (orange) is composed of 13–14 β-strands with a conserved autoproteolytic cleavage triad (GPS) of sequence HL | T/S (red triangle: cleavage site), followed by the tethered agonist (TA, yellow). **c** the GAIN domain of rat ADGRL1 (PDB ID: 4DLQ^9^) shows all hallmarks of the GAIN domain.

Adhesion/class B2 G protein-coupled receptors (aGPCRs), the second-largest class of GPCRs, have garnered substantial research and medical interest due to their involvement in neural development, hereditary disorders, and cancers among others^1–4^. aGPCRs are classed into nine subfamilies^5^ and are characterized by a very large extracellular region, containing the conserved GPCR autoproteolysis inducing (GAIN) domain. The GAIN domain is positioned directly N-terminal of the seven-transmembrane domain (7TM, Fig. 1a), which transduces an extracellular signal to intracellular effector proteins^6^.

The GAIN domain serves several functions. First, at its GPCR proteolysis site (GPS) an autoproteolytic cleavage event occurs adjacent to the 7TM domain, which yields a bipartite structure stabilized by non-covalent interactions^7–10^. The two resulting elements, called N-terminal and C-terminal fragments (NTF/CTF), remain attached to one another even at the cell surface. Second, the GAIN domain contains a tethered agonist element (TA, Stachel, Fig. 1b), which corresponds to the N-terminus of the CTF that arises through GAIN domain cleavage^11–14^. The TA activates the receptor upon dissociation of the NTF/CTF complex^11,12,15–17^, the biophysical intricacies of which are yet to be uncovered^18,19^. Third, several aGPCRs act as metabotropic mechanosensors^20–24^, where the GAIN domain is proposed to serve as a molecular integrator of mechanical forces through its partial unfolding or eventual dissociation of the NTF/CTF complex upon force stimulation^25–29^.

X-ray and cryo-EM structures provided the first insights into GAIN domain structures and TA-7TM complexes^9,10,30–36^. The available set of experimentally determined GAIN domain structures indicates a common architecture with two, structurally variable, subdomains: The more variable subdomain A is comprised of up to six helices, and the more sequence-conserved subdomain B adopts a β-sandwich with the TA as its most C-terminal strand (Fig. 1b, c)^9,10,19,35–37^. The low sequence identity of GAIN domains and variable number of constituting segments, however, led to inadequate annotations of the GAIN domain in protein databases, hampering inter-species comparison of GAIN domains and limiting a holistic understanding of GAIN domain function.

Generic residue numbers (GRNs) provide a common index to corresponding amino acids across the different members of a protein family. GRNs have a great utility as they enable comparison and inference of a multitude of residue data spanning pharmacology (e.g., in vitro mutations), structural biology (e.g., ligand, domain, or protein interactions), and genetics (natural variants). For GPCRs, the first GRN scheme was that of *Ballesteros-Weinstein* and assigned residue indices in the 7TM domains of class A GPCRs^38^. A number, 50, is given to the most conserved residue in each of the seven helices that serve as a reference when assigning consecutive numbers of upstream and downstream. This system has since been adapted to other GPCR classes^39^, including the Wootten numbering scheme for the class B1 (Secretin) and B2 (Adhesion) receptor families^40^.

As GPCR structures became available, these sequence-based schemes were found to suffer from non-generic numbers when some receptors have helix bulges or constrictions causing a one-position residue gap in structural alignment and offset of following residues in sequence alignment^39,41^. To mitigate this numbering issue, the GPCR database (GPCRdb) provided structure-based GRN schemes for each GPCR class wherein structural residue gaps are also present as single gaps in the sequence alignment^39^. The GPCRdb schemes also added helix 8 (H8) and structurally conserved stretches of the first extra- and intracellular loops. Highly flexible and variable protein regions such as loops remain unannotated in currently established schemes. GRNs have seen wide adoption among researchers, becoming a frequently used tool in communicating GPCR research. Other GRN schemes like the kinase−ligand interaction fingerprints and structure database (KLIFS)^42–44^, the common G protein Gα numbering (CGN)^45^, or the common arrestin numbering (CAN)^46^ serve in mapping functional protein networks or drug-development purposes in additional protein families.

Here, we introduce a GRN scheme for aGPCR GAIN domains based on the superposition of more than 14,000 structural GAIN domain models generated with ColabFold/AlphaFold 2^47,48^. We highlight structural variability and common features of all aGPCR GAIN domains and enable data transfer by means of common GRN labels by finding statistically cancer-enriched positions in humans. The GAIN-GRN was implemented into the GPCRdb^41,49–51^ to allow for intuitive use in a highly accessible and widely adapted resource, as well as for programmatic access to the data. In addition, we provide a notebook in the code repository (https://github.com/FloSeu/GAIN-GRN) enabling the ad-hoc indexing of any GAIN-domain containing protein. We expect that these will inspire future studies aiming to elucidate the molecular mechanism of GAIN domains in the signal transduction and physiological functions of aGPCRs, and will aid analyses on how structural anomalies contribute to aGPCR dysfunction under disease conditions.

## Results

### The heterogeneity of the GAIN domain necessitates structure-based residue numbering

A comprehensive analysis of GAIN domains by means of multiple sequence alignments fails due to their low sequence identity and variable number of segments (α-helices and β-strands)^9,10,35,36^. Thus, to enable a comprehensive description of the GAIN domain, we opted for a structure-based approach, for which we generated a set of 14,435 GAIN domain models encompassing orthologs of the 33 mammalian aGPCR and 916 GAIN domains not matched to any orthologs with ColabFold/AlphaFold 2^47,48^. In order to assess the composition of both GAIN subdomains, we used structural alignments with GESAMT^52^ for indexing segments. Using this approach, the segment position in space determines its index instead of its sequence-based order, allowing the assignment of equivalent positions for GRN indexing in the context of variable domain composition.

**Fig. 2:**
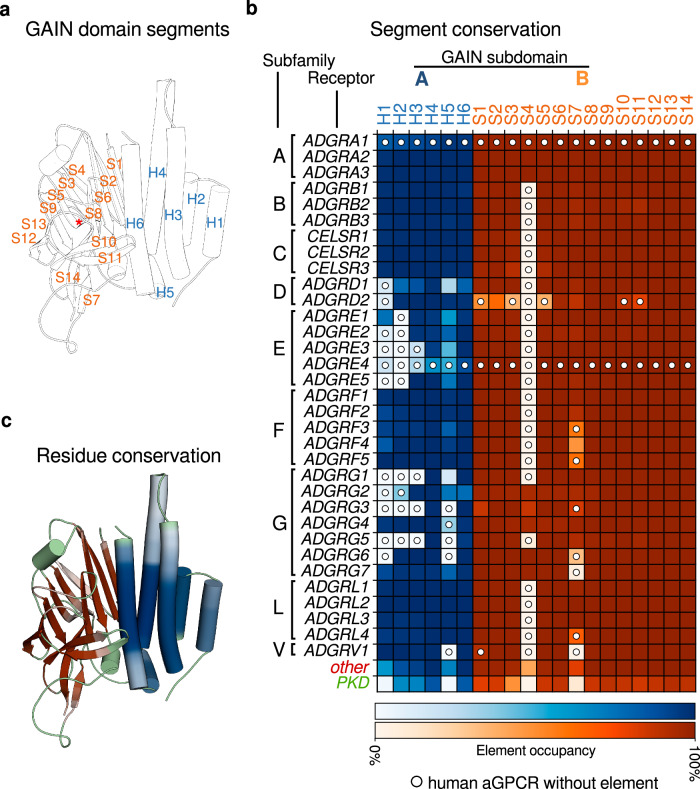
GAIN domain helix and strand architecture of human adhesion G protein-coupled receptors (aGPCRs) and conservation across orthologs. **a** Example chimeric GAIN domain structure showing all six helices, 14 strands, and the GPCR proteolytic site (GPS) on its topology, a red asterisk marks the autoproteolytic cleavage site N-terminal of the tethered agonist (S14). **b** each row represents all orthologs in the UniProtKB database that have a GAIN domain for each receptor, where individual elements are highlighted by occupancy (blue: subdomain A helices, orange: subdomain B strands), higher color intensity represents a higher conservation of the element within the group of orthologs. White circles denote elements that are not present in the corresponding human aGPCR GAIN domain (ADGRA1 without GAIN; ADGRE4 is a pseudogene in humans). Other receptors (red label) are aGPCRs without a receptor ortholog in humans. A set of 2872 polycystic kidney disease-type proteins (PKD, green label) have GAIN domains, which were matched against the set of aGPCR templates, matching well with an additional beta-sandwich subdomain between extended S9 and S10. **c** residue conservation for residues indexed with the GAIN-GRN for subdomain A (shades of blue) and subdomain B (shades of orange), with 14435 GAIN domains as the underlying number of GAIN domains. Unindexed residues are colored green.

Based on the complete set of GAIN domain models, we asserted that subdomains A and B are composed of two to six helices and 12–14 strands, respectively, which we indexed using the identifiers H1-6 and S1–14 (Fig. 2a). Subdomain B exhibits generally high segment conservation, with only strand 4 specific to subfamilies A and G (Fig. 2b). The composition of subdomain A is more variable. While the A, B, C, F, and L subfamilies all have six helices, the D and G subfamilies show heterogeneity ranging from two to six helices (Fig. 2b). Structures reflecting subdomain A variability are for example the rat ADGRL1 with a six-helix bundle (Fig. 1c)^9^, and the human ADGRG1 GAIN domain with only Helix 4 and 6^36^, the two most conserved helices in the dataset.

When looking at individual residue positions (to which GRN labels are assigned, Fig. 2c), notably the center regions of helix 3, 4, and 6 are more frequently occupied (occupancy referring to the fraction of models containing a segment in the dataset) than the extreme positions, highlighting varying helix lengths in the model dataset, with especially the L subfamily exhibiting longer helices. Aside from the residues in the less conserved strands 4 and 7, there is high occupancy in subdomain B. The unindexed GAIN domain loops connecting the structured elements show very different lengths, frequently exceeding 50 residues (Supplementary Fig. 6). Notably, a total of 84 homologs of ADGRA1 in 47 species have a GAIN domain, which is not found in human ADGRA1^9^, whereas 78 GAIN domains were identified for ADGRE4, which is a pseudogene in human (Supplementary Table 1).

### Generic residue numbering denotes corresponding GAIN domain amino acids across receptors

**Fig. 3:**
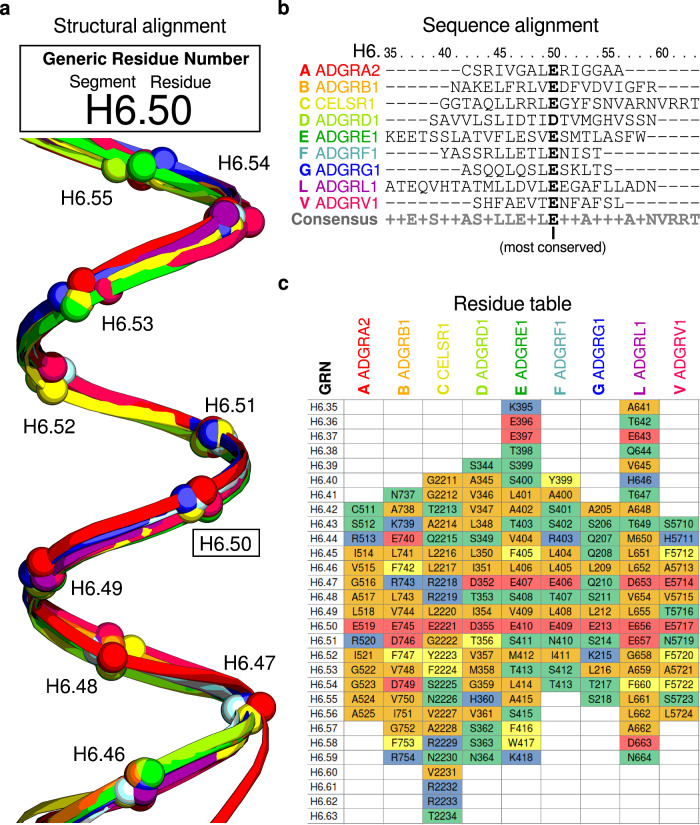
Generic residue numbering denotes corresponding residues across receptors. Generic residue numbers (GRNs) are equivalent residues in structure and sequence across receptors, enabling comparisons of, e.g., mutations^39,53,85^, sequence conservation^39^ structural contacts, and ligand interactions^50,85^. **a** A GRN is composed of the segment identifier and the numeric residue index. The structural alignment of the GAIN helix 6 segment of nine human adhesion G protein-coupled receptors indicates the GRN at the respective aligned residue Cα (spheres). **b** sequence alignment of the nine receptors with the receptor subfamily indicated in bold in front of the receptor name. Sequence alignment is based on structural alignment, with the H6.50 GRN denoting the most conserved residue in the segment (bold). All other segment residues are indexed relative to the .50 position. **c** residue table of aligned segments colored by chemical properties.

Based on the GAIN-GRN indexing of all 14,435 structural models, we created comprehensive alignments of the GAIN domain in structure and in sequence (Supplementary Fig. 1). These provide a novel utility to map data across all adhesion-GPCRs and cross-map sequence-position specific data between homologs. A schematic of the GRN assignment process is outlined in Fig. 3. Each GRN consists of the segment identifier (e. g. Helix 6 = “H6”) and the respective index relative to the most conserved residue in the segment, separated by a dot (e.g., “H6.50”). In this example, representative GAIN domains from each aGPCR subfamily are structurally aligned, with the Cα-atoms corresponding to GRN positions (Fig. 3a). Using the backbone alignment positions as the basis for a sequence alignment, the most conserved position is identified here as the acidic E/D and gets assigned the “center” .50 index (Fig. 3b). A residue table of the aligned segments highlights variation in segment lengths and reveals positions with similar physicochemical properties, e.g., the H6.50 as acidic, H6.45, H6.46 and H6.49 as aliphatic, despite low sequence identity (Fig. 3c).

### GPCRdb resources aiding use of GAIN GRNs

**Fig. 4:**
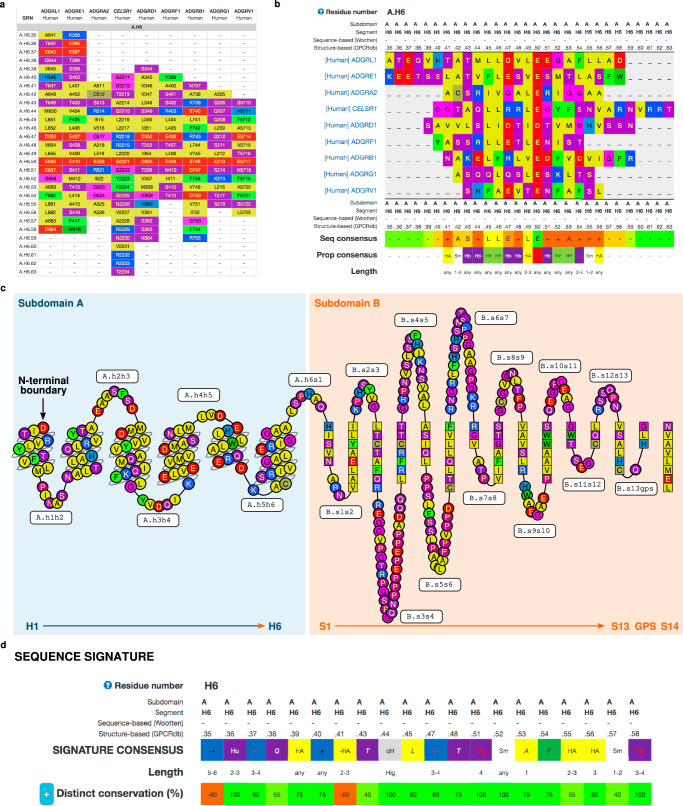
GAIN-GRN data and tools available in the GPCR database, GPCRdb. **a** Generic residue numbering tables show GRNs followed by receptor-specific residue numbers and amino acids^53^. **b** Sequence alignments allow swift sequence comparison across all GAIN segments (helices and strands) as well as conservation (% identify and consensus sequence) and physicochemical properties (residue polarity, size, helical propensity and z scales)^50^. **c** the snake plot of the GAIN domain provides a simple 2D representation with the option of custom coloring^53^. **d** The Sequence signature tool identifies structural determinants – uniquely conserved residues – upon contrasting two sets of sequence alignments of receptors that have and lack the given function, respectively^50^.

As part of the GPCRdb integration, we assigned the 6 helices (H1-6), 14 strands (S1–14), and 21 loops connecting segments (h1h2, h2h3, h3h4, h4h5, h5h6, h6s1, s1s2, s2s3, s3s4, s4s5, s5s6, s6s7, s7s8, s8s9, s9s10, s10s11, s11s12, s12s13, s13gps, gpss14, s14tm1) to all Class B2 (Adhesion) sequences. In addition, the GPS motif has a separate assigned segment from the conventional GPS.-2, GPS.-1, and GPS. + 1 notation for the three residues directly preceding or following the catalytic site. With the introduction of these segments, researchers can apply the GPCRdb toolkit to the whole, or selected parts of the GAIN domain (Fig. 4). We updated the snake plots of the Class B2 (Adhesion) GPCRs to contain the segments of the GAIN domain (Fig. 4c). The snake plots can be found on the Receptor page (https://gpcrdb.org/protein/) with custom coloring options^53^. Along with the already provided data, GAIN domain data is also accessible programmatically via REST API, enabling seamless integration of GAIN-GRN into Python workflows with, i.e., the mdciao package^54^.

### Consensus contacts stabilizing the GAIN domain fold

Addressing GAIN domain positions via GRN enables mapping any GRN-label-dependent information across aGPCR homologs. Corroborating the analysis of tertiary structures, we exploit the GRN indexing to consolidate pairwise residue-residue contacts - particular to each structure - into unified consensus contacts. The entirety of GRN-label pairs occurring over the complete dataset with a given frequency yields the GAIN domain contactome, shown in Supplementary Fig. 7 as a flareplot. This plot represents a contact matrix, individually resolving consensus contacts at the residue level while highlighting contact relationships between the different GAIN segments.

The importance of H6 as a “hub” connecting the subdomains A and B is clearly visible with highly conserved contacts to primarily S6 and also S2, S8, S10, and S14. Furthermore, H4 partially tethers subdomains A and B via highly conserved contacts to S1 and S2. In Supplementary Tables 2 and 3, the most frequent inter-domain and GPS contacts are listed individually, respectively. In addition, we coarse-grained the contactome into the GAIN segments (Supplementary Fig. 8) reproducing the tethering structure of the segments regardless of individual contacts, further highlighting H4 and H6 as the segments mediating contacts between both GAIN subdomains.

### A map of cancer-enriched mutations in the GAIN domain

**Fig. 5:**
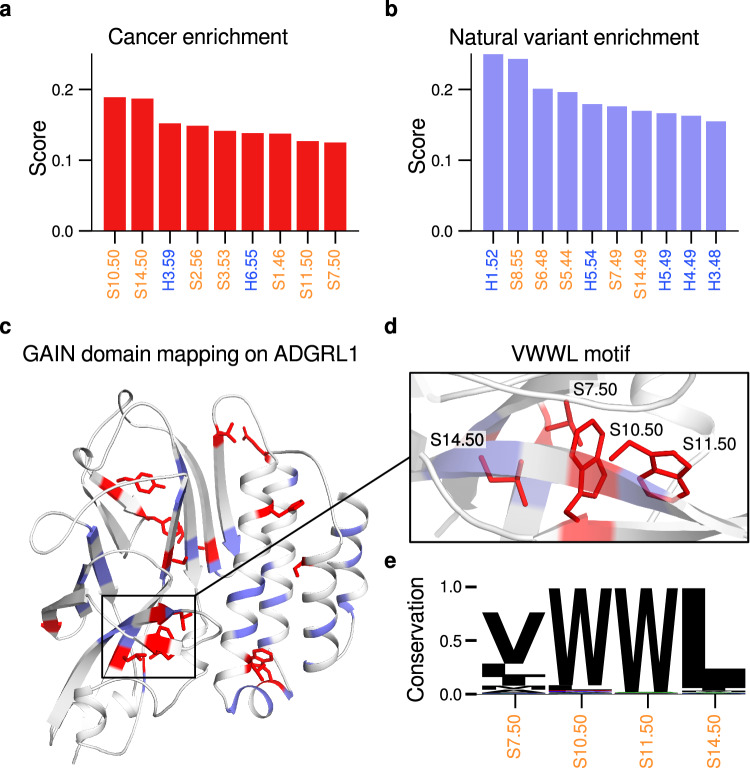
Cancer Genome Atlas mutations mapped onto the GAIN domain via Generic Reisdue Numbers show mutational hotspots. Enrichment scores were calculated via a relation between the number of naturally occurring variants (retrieved from Genome Aggregation Database (gnomAD), accessed on Jan 16^th^, 2023 from https://registry.opendata.aws/broad-gnomad)^86^. and cancer-associated mutations (retrieved from The Cancer Genome Atlas (TCGA) Genomic Data Commons, (GDC), portal.gdc.cancer.gov)^87^ at a GRN according to the formula from Wright et al., 2019^57^. **a** The ten most cancer-enriched positions in humans, with the S4 element excluded due to too low a number of mutations and variants. **b** The ten most natural variant enriched positions, showing negative association with cancer, analogous to the mutation enrichment score^57^ **c** enriched positions mapped onto the human ADGRD1 GAIN domain model (UniProt ID: Q6QNK2), with values above 0.1 of maximum intensity colored for cancer enriched (red sticks) and variant enriched (lavender) positions. **d** a cluster of cancer-enriched positions shows the most conserved residues of four strand segments (S7.50, S10.50, S11.50, and S14.50) contacting each other. All positions are part of the ten most cancer-enriched positions. **e** logo plots of residue conservation (fraction of total structures with 1.0 meaning that the position is conserved in all 14,435 GAIN domains in the dataset) for the enriched cluster show strong residue conservation for the VWWL motif composed of V^S7.50^, W^S10.50^, W^S11.50^, L^S14.50^.

The GAIN domain, present in 31 of 32 human aGPCRs, is a mutational hotspot affected by various pathologies^7,9,55,56^. To find potential cancer-enriched positions and differentiate them from variance-enriched positions, we adopted the cancer-enrichment score from Wright et al.^57^ for all 31 human aGPCR GAIN domains (Fig. 5a, b) indexed by the GAIN-GRN. While generally, enriched positions of both types are distributed throughout the GAIN domain (Fig. 5c), eight of the ten most cancer-enriched GRN positions are found in subdomain B carrying the TA (Fig. 5a and Supplementary Fig. 2).

We identify a “VWWL” motif consisting of four conserved top-ten cancer-enriched residues S7.50, S10.50, S11.50 and S14.50. This buried motif is located in the direct vicinity of the GPS cleavage site (Fig. 5d–e and Supplementary Fig. 1), with mutations known to affect GAIN domain autoproteolysis and TA function: the conserved leucine at the S14.50 position (Supplementary Fig. 1) is a TA residue deeply buried into the orthosteric binding site of the 7TM domain in active aGPCR-7TM structures^19,30–34,58^, and its mutation led to altered receptor activity^11,22,59,60^. Moreover, the mutation of any tryptophane within the “VWWL” motif causes loss of function in rat ADGRL1^9^.

With a comprehensive analysis of human aGPCR GAIN domains, we find a total of 46 statistically cancer-enriched positions (Supplementary Fig. 2). By using the GAIN-GRN, homologous cancer-enriched residue positions can now be assigned to any GAIN domain and analyzed for their individual origin and consequence with the tools provided in the repository. This allows the transfer of positional information between GAIN domains in different species, particularly from humans to model organisms, such as *D. melanogaster*, *C. elegans* or *D. rerio*^61–64^. The functional analyses in these model systems can now provide valuable insights into the molecular causes of cancer mutations in humans in future studies.

To further highlight the potential of our approach to evaluate position-specific information, we applied our code repository to conduct a more in-depth sequence and structural bioinformatics analysis of the natural variants neighboring the VWWL motif. The side chains of two natural variance-enriched S7.49 and S14.49 positions, neighboring the cancer-enriched x.50 residues of the VWWL motif point away from the cleavage site. At S7.49 a manifold of amino acid substitutions is found in agreement with its unburied position. By contrast, at the buried S14.49 position, we exclusively find substitutions to hydrophobic residues (Supplementary Fig. 9). Due to solvent exposure the position S7.49 accordingly seems rather insensitive to sequence variations, while at S14.49 variations to non-hydrophobic residues seem not to be tolerated.

### GAIN domains of PKD1/PKD1-like proteins possess an extended topology

The only other protein family known to contain GAIN domains are PKD (polycystic kidney disease)1/PKD1-like proteins (in short here PKD1; also referred to as polycystin-1[PC1])^9^. Mutations in PKD1 are responsible for the majority of autosomal dominant kidney PKD, a devastating disorder that entails the development of cysts in the kidney and other organs leading to their eventual failure^65^. PKD1 GAIN domains display similar molecular properties as aGPCR GAIN domains with autoproteolytic cleavage resulting in a bipartite NTF-CTF protein layout after proteolysis^66^. Enabling the comparison and transfer of experimental and mutational knowledge between aGPCR and PKD1 GAIN folds is the basis for the understanding of similarities and differences between the two, and can offer valuable insights into the cell biological and physiological consequences of GAIN domain functions. However, thus far such transfer has been obstructed by the lack of clear homology assignments of primary and secondary structural positions between aGPCR and PKD1 GAIN domains.

Here, we employed the GAIN-GRN scheme to allocate positional labels in PKD1 GAIN domains and compare them to those of aGPCR GAIN folds. Since no experimental structure of PKD1 GAIN domains is available yet, we prepared 2738 structural models analogously to the aGPCR dataset^66–68^. We applied the GAIN-GRN scheme to the models, which on average resulted in four subdomain A helices and twelve subdomain B strands recognized by the GAIN-GRN method (Fig. 2b), thus structural elements homologous to aGPCR GAIN domains. Interestingly, we also observed differences to aGPCR GAIN domain layouts as the PKD1 GAIN domains showed an additional β-sandwich fold, which contains an extension of S10 and C-terminally elongated TA. Finally, we also observed up to a total of eight subdomain A helices with additional, unindexed helices (Supplementary Fig. 3).

In sum, the GAIN-GRN scheme provides a robust strategy to allocate structural homologies at the primary and secondary levels also to GAIN folds of PKD1 molecules, which now renders positions in both GAIN domain types comparable to one another.

## Discussion

The GAIN domain is an ancient extracellular protein domain of the large adhesion GPCR family, involved in neural development, hereditary disorders, and cancer^1–4^. Despite recent insights obtained from high-resolution structures of GAIN domains in complex with the 7TM^18^, the GAIN domain function in autoproteolysis, mechanosensing, and TA-dependent receptor activation is still poorly understood. To overcome the limitations imposed by the structural heterogeneity of GAIN domains in aGPCR homologs - the variable number of secondary structure segments and overall low sequence identity - we developed the GAIN-GRN as a generic residue numbering scheme for aGPCR GAIN domains. We used spatial alignments of structural models, generating multiple sequence alignments to define the reference residue position as the most conserved residue in each segment^39,50,53^. The GAIN-GRN is based on GAIN domain models predicted by AlphaFold 2/Colabfold to include most GAIN domains in proteins present in the Uniprot database^42,43^. To aid users in employing GAIN GRNs for data analysis and hypothesis generation, we implemented the GAIN-GRN in the GPCRdb serving as an accessible and established resource (Fig. 4). We also show that the GAIN-GRN is a robust tool to assign structure-homologous residues across molecule families as we have retrieved GRN also for PKD1 GAIN domains. Upcoming experimental structures of GAIN domains may have constrictions or other unpredicted structural features that would require future refinement of the GAIN-GRN algorithm, which would be implemented by a new set of structural templates. Notably, the structure-based statistical approach of generating generic residue numbers may also be generalizable by making appropriate adjustments to the source code as outlined in the provided code repository (https://github.com/FloSeu/GAIN-GRN).

Statistical evaluation of the dataset of aGPCR GAIN domains enables us to assess their composition as well as the spatial and positional conservation of information as reflected by the GAIN *contactome* (Supplementary Figs. 7, 8). The evolutionarily conserved two-subdomain architecture of the GAIN domain is present in humans as well as distantly related organisms such as *Trichoplax adhaerens*^5,69–72^. Subdomain B, containing the autoproteolytic cleavage site and the tethered agonist, is structurally less variable consisting of 12–14 β-strands, in agreement with its implied function in NTF-CTF association, force-dependent GAIN domain separation and mechanosensing^26,27,29^. Notably, our analysis underlines the notion that the known “GPS motif” is not an individual protein domain, as initially anticipated, but rather the C-terminal section of subdomain B^8,9^. By contrast, subdomain A, shows high structural heterogeneity with only two critically conserved helices (H4 and H6, Fig. 2), with their core regions forming an interface with and presumably stabilizing subdomain B (Supplementary Figs. 7, 8). Despite structural heterogeneity, our structure-based alignment reveals highly conserved stretches of residues and segments with low overall sequence identity but similar physicochemical properties (Supplementary Fig. 1), thus corroborating the notion that structural conservation outweighs sequence conservation^73^.

Creating structure-based alignments of larger protein sets with representatives in humans enables us to structurally map benign and malign mutations for testing in homologous positions of distantly related proteins. For example, the mutations within the “VWWL” motif (Fig. 5) close to the GPS may now be tested in any model system based on their GRN index. Analogously, we can now assess the location of known pathological mutations: Avila-Zozaya et al. have investigated cancer-related mutations in ADGRL3, with impacts on G_13_-signaling for K561N^H1.51^, D798H^S9.47^, S810L^s9s10^ and E811Q^s9s10^, where the latter two residues correspond to the interaction region of the GAIN domain with the seven-transmembrane domain^19,56^. Two mutations responsible for loss of surface expression in GPR56, causing bilateral frontoparietal polymicrogyria (BFPP), are the highly conserved C346S^S10.47^ and W349S^S10.50^ refs. ^7,74,75^ More generally, our approach promotes future experiments focusing on the central role of GAIN domains in the physiological functions of aGPCRs and PKD1 molecules and will aid analyses of how structural anomalies contribute to their dysfunction under disease conditions.

## Methods

All computational pipelines were implemented in Python 3.9.16. Packages used for data collection: colabfold 1.2.0, alphafold-colabfold 2.1.6, docker 5.0.3. Packages used for data analysis: stride, ccp4.8.0 with GESAMT 8.0, numpy 1.23.2, matplotlib 3.7.1, Jupyter 1.0.0, Ipykernel 6.19.2, Logomaker 0.8, nglview 3.0.4, Scikit-learn 1.3.0, colabfold 1.2.0, pandas 1.5.3, MAFFT 7.490. Figures were created with The PyMOL Molecular Graphics System, Version 2.2.5, Schrödinger, LLC.

### Generation of the GAIN domain model dataset

Sequences were retrieved from the UniProtKB database with two queries for adhesion GPCR and CELSR, respectively, yielding 22,946 and 2179 sequences, respectively (Supplementary Fig. 5). Sequences were filtered for a minimum length of 50 residues and the presence of a “GPS” domain annotation in their domain records. The C-terminal sequence boundary was read from the “GPS” Domain record, whereas for the N-terminal boundary, sequence lengths exceeding 800 residues were truncated to include the GAIN domain boundary expected at around 320–360 residues upstream of the C-terminal boundary, resulting in 16,537 sequences. The structures of all processed sequences containing potential GAIN domains were predicted with ColabFold^47,48^ by using batches of 30 length-sorted sequences with a pre-defined padding to account for sequence length differences per batch Root mean square deviation (RMSD) values of GAIN domains from a respective experimentally determined structure were generally low, with PDB: 4DLQ, rat ADGRL1, RMSD 0.45 Å; PDB: 4DLO, human ADGRB1, RMSD 1.34 Å; PDB: 5KVM, human ADGRG1, RMSD 0.77 Å; PDB: 6V55, danio rerio ADGRG6, RMSD 0.82 Å; PDB: 8IKJ, human ADGRE5, RMSD 0.98 Å; PDB 7QU8, human ADGRG3, RMSD 0.96 Å), and deemed sufficient to keep the modeled structures instead of the experimental ones. A multiple sequence alignment was constructed from an initial 15,957 successfully folded and non-doublet aGPCR/CELSR sequences using MAFFT^76^ for localizing the GPS motif.

The secondary structure information of the resulting folded structures was read out with STRIDE^77^ The data from the resulting files was used to apply two criteria for a valid GAIN domain: The presence of both the helical subdomain A and the β-sandwich subdomain B as well as the existence of the GPS or a homologously aligned sequence. The filtered dataset consists of 14,435 valid GAIN domains (Supplementary Fig. 5). The human dataset consists of 31 aGPCR GAIN domains.

### GAIN domain detection

The presence of both subdomains was detected by using a numerical transformation of the sequence, assigning a 1 to helical and − 1 to β-strand residues. By using linear convolution, a signal was generated, whose sign changes were detected as boundaries between helical and β-strand protein segments. The presence of both subdomains was confirmed by identifying the largest helical segment adjacent to a C-terminal β-strand segment corresponding to the subdomain in each respective structure. The signal decay N-terminal of subdomain A, by the presence of non-helical residues, was used to determine the GAIN domain boundary for each generated model. The column index of the GPS.-1 residue (corresponding to leucine in the conserved HL | S/T triad) was set as a reference for detecting the presence of a GPS motif or homologous aligned sequence elements. Any structure showing a residue at the corresponding column in the MSA was set as possessing the GPS, therefore satisfying the second criterion.

### Template model selection

Template candidates were extracted by selecting a random 400 structures of each subfamily GAIN domain model to account for variance in the selection and optimize performance. The sub-selection was used to generate a root-mean-square deviation (RMSD) matrix by pairwise alignment applying GESAMT in the CCP4.8 package^52,78^, in pairwise alignment mode, on the respective subdomains. Since pairwise alignments form the basis of the segment identification process and creation of the following MSA, only the pairwise mode of GESAMT was used for code consistency. The matrix was clustered and sorted using agglomerative clustering via the scikit-learn python package^79^, and the lowest-RMSD model of the largest cluster was selected as a candidate template. Candidate templates were checked against each receptor sub-selection of the dataset via occupancy (fraction of structures matching the template anchor) and distance (pairwise Cα-Cα distance). Repeating the template selection and curating workflow four times, matched receptors of low quality from the initial set were removed, and additional templates were added and selected from individual receptor selections. After accounting for all conflicts with the alignment functionality in GPCRdb, coverage of all aGPCR protein paralogs, and coverage of spatial orientation variance in individual segments, manual adjustments were made in the form of including strand 4 of subdomain A with 10% occupancy in the model dataset, and an adjustment of the center position in helix 4 to account for the variance in orientation in helix 4, sometimes overlapping with Helix 5. The final set of templates consisted of 15 subdomain A and two subdomain B templates for the complete indexing. Segment center residues for each element were generated by pairwise aligning all GAIN domains against each candidate template using GESAMT and collecting all pairwise residue matches into a multiple sequence alignment, finding the position of highest occupancy and residue identity. Segment centers were validated and manually curated via 3-D, aligning all candidate templates and verifying the identical position of the anchor in space. The position of the H4 segment center was manually adjusted to avoid ambiguities with the H5 residue center. The unique orientation of the most N-terminal helix of ADGRD1, ADGRE1, and ADGRF4 yielded three individual segment centers. Each receptor GAIN was assigned a template per subdomain to be matched to by default.

### Segment overlap and ambiguity cases

For some cases of low-quality proteins, the SSE of the template and GAIN were overlapping without a pairwise match of the template anchor. In these cases, the match closest to the template anchor was set as the reference position considering the offset (i.e., “S14.47” when the residue is three residues N-terminal of the template S14.50) and enumerated analogously from there.

Anchor ambiguity cases arose when two elements were detected as one by STRIDE with two template center residues matched, however the spatial orientation of two SSE was distinguishable. These cases were handled by a hierarchical segment splitting routine assessing the segment between both matched segment centers in decreasing priority: the presence of a coiled residue, a residue with backbone angles outside of five standard deviations of the element total distribution in the dataset, presence of proline or glycine and a manually defined truncation element for common occurrences.

### Creating the template set

Templates are defined as consensus structural models used for structural alignment of other GAIN domain models for segment identification and indexing. Templates were defined separately for GAIN subdomains A and B. The definition of the template set consisted of three steps: Identifying candidate template structures, finding their center positions, and assessing their coverage and quality for integration into the final template set (Supplementary Fig. 4). Templates have the center residues of each segment already assigned based on structural alignments of all template structures (Fig. 3b).

### Indexing via GAIN-GRN

Each GAIN domain was pairwise aligned to its assigned subdomain A and B template, respectively. GAIN domains not assigned a receptor were structurally aligned to all templates using GESAMT^52^, selecting the lowest RMSD template for each subdomain. For each SSE the residue matching the template center was labeled “##.50” with the corresponding element name (H1–H6, S1–14), enumerating all residues in the SSE with numbers decreasing in the N-terminal and increasing in the C-terminal direction. Each ordered residue in the GAIN was assigned a label and exported tabulated. An additional workflow was created in an interactive notebook enabling the assignment of the GAIN-GRN for any protein with an associated model in the alphafoldDB^80^ either retrieving the information about the GPS from the Uniprot database or manually defining the C-terminal GAIN boundary.

### Mutation mapping onto GRN positions

Mutations were retrieved from the Cancer Genome Atlas (TCGA, within the Genomic Data Commons https://portal.gdc.cancer.gov/) for each of the 31 human aGPCRs, yielding a total of 6874 individual mutations. A routine was implemented to correct the residue indices of the GAIN domain residues to match the UniProtKB indices. By matching each position, we assigned the GRN to each occurring mutation within the indexed GAIN domain space with a total of 861 mutations, of which 769 mutations were within ordered segments with individual labels. In addition, we implemented a parsing routine to parse the mapped mutations, map the number of mutations and their occurrence onto any GRN-mapped GAIN domain, and filter mutations by the impact metrics SIFT and Polyphen^81,82^ to tailor the query routine to the individual purpose. In our example, cancer-enriched positions were extracted by calculating the number of cancer-associated mutations against the number of natural variants extracted from dbSNP (www.nbci.nlm.nih.gov/snp/) analogous to Wright et al., 2019^57^.

### Contact frequencies

For each of the 14,435 GAIN domain structures in the dataset, heavy-atom residue-residue contacts were computed using a distance cutoff of 4 Å. All pairs of residues sharing a contact are aggregated into a single contact matrix which is indexed with GRN labels. Some elements of this matrix are shown partially as well as in full in Supplementary Tables 2, 3, and Supplementary Data 1. The computation of contacts, GRN label-handling, and plotting (flareplot and contact-matrix) was done using mdciao^54^.

### Reporting summary

Further information on research design is available in the Nature Portfolio Reporting Summary linked to this article.

## Supplementary information

Supplementary Information

Description of Additional Supplementary Files

Supplementary Data 1

Reporting Summary

Transparent Peer Review file

## Source data

Source Data

## Data Availability

The generated GAIN domain models generated in this study, alongside all analysis data needed for re-producing all data during the process outlined in the methods have been deposited in the online repository zenodo under accession code 12515545^83^. The PDB entries used in this study are available under the following accession codes: 4DLQ, 4DLO, 5KVM, 6V55, 8IKJ, 7QU8 The UniProtKB entries used in this study can be accessed under the following accession code Q6QNK2 Source data are provided in this paper.

## Code availability

The generated code and interactive notebooks are available under https://github.com/FloSeu/GAIN-GRN and also accessible in the online repository zenodo under accession code 14140353^84^.

## References

[1] Chiang, N.-Y. et al. GPR56/ADGRG1 Activation promotes melanoma cell migration via NTF dissociation and CTF-mediated Gα12/13/RhoA signaling. *J. Invest. Dermatol.* **137**, 727–736 (2017). 27818281 10.1016/j.jid.2016.10.031

[2] Scholz, N. Cancer cell mechanics: Adhesion G protein-coupled receptors in action? *Front. Oncol.* **8**, 59 (2018). 29594040 10.3389/fonc.2018.00059 PMC5859372

[3] Kan, Z. et al. Diverse somatic mutation patterns and pathway alterations in human cancers. *Nature* **466**, 869–873 (2010). 20668451 10.1038/nature09208

[4] Langenhan, T., Piao, X. & Monk, K. R. Adhesion G protein-coupled receptors in nervous system development and disease. *Nat. Rev. Neurosci.* **17**, 550–561 (2016). 27466150 10.1038/nrn.2016.86

[5] Wittlake, A., Prömel, S. & Schöneberg, T. The evolutionaryhistory of vertebrate adhesion GPCRs and its implication on their classification. *Int. J. Mol. Sci.* **22**, 11803 (2021). 34769233 10.3390/ijms222111803 PMC8584163

[6] Batebi, H. et al. Mechanistic insights into G-protein coupling with an agonist-bound G-protein-coupled receptor. *Nat. Struct. Mol. Biol*. **31**, 1692–1701 (2024). 10.1038/s41594-024-01334-2 PMC12132790 38867113

[7] Prömel, S., Langenhan, T. & Araç, D. Matching structure with function: The GAIN domain of Adhesion-GPCR and PKD1-like proteins. *Trends Pharmacol. Sci.* **34**, 470–478 (2013). 23850273 10.1016/j.tips.2013.06.002

[8] Liao, Y., Pei, J., Cheng, H. & Grishin, N. V. An ancient autoproteolytic domain found in GAIN, ZU5 and Nucleoporin98. *J. Mol. Biol.* **426**, 3935–3945 (2014). 25451782 10.1016/j.jmb.2014.10.011 PMC4258472

[9] Araç, D. et al. A novel evolutionarily conserved domain of cell-adhesion GPCRs mediates autoproteolysis. *EMBO J.* **31**, 1364–1378 (2012). 22333914 10.1038/emboj.2012.26 PMC3321182

[10] Pohl, F. et al. Structural basis of GAIN domain autoproteolysis and cleavage-resistance in the adhesion G-protein coupled receptors. Preprint at 10.1101/2023.03.12.532270 (2023). 10.1038/s41467-026-71225-1 PMC13062082 41942442

[11] Liebscher, I. et al. A tethered agonist within the ectodomain activates the adhesion G protein-coupled receptors GPR126 and GPR133. *Cell Rep.* **9**, 2018–2026 (2014). 25533341 10.1016/j.celrep.2014.11.036 PMC4277498

[12] Stoveken, H. M., Hajduczok, A. G., Xu, L. & Tall, G. G. Adhesion G protein-coupled receptors are activated by exposure of a cryptic tethered agonist. *Proc. Natl Acad. Sci. USA* **112**, 6194–6199 (2015). 25918380 10.1073/pnas.1421785112 PMC4434738

[13] Mathiasen, S. et al. G12/13 is activated by acute tethered agonist exposure in the adhesion GPCR ADGRL3. *Nat. Chem. Biol.* **16**, 1343–1350 (2020). 32778842 10.1038/s41589-020-0617-7 PMC7990041

[14] Zhu, B. et al. GAIN domain-mediated cleavage is required for activation of G protein- coupled receptor 56 (GPR56) by its natural ligands and a small-molecule agonist. *J. Biol. Chem.* **294**, 19246–19254 (2019). 31628191 10.1074/jbc.RA119.008234 PMC6916468

[15] Paavola, K. J., Stephenson, J. R., Ritter, S. L., Alter, S. P. & Hall, R. A. The N terminus of the adhesion G protein-coupled receptor GPR56 controls receptor signaling activity. *J. Biol. Chem.* **286**, 28914–28921 (2011). 21708946 10.1074/jbc.M111.247973 PMC3190698

[16] Frenster, J. D. et al. Functional impact of intramolecular cleavage and dissociation of adhesion G protein–coupled receptor GPR1. *J. Biol. Chem.* **296**, 100798 (2021). 34022221 10.1016/j.jbc.2021.100798 PMC8215292

[17] Yang, L. et al. GPR56 Regulates VEGF production and angiogenesis during melanoma progression. *Cancer Res.* **71**, 5558–5568 (2011). 21724588 10.1158/0008-5472.CAN-10-4543 PMC3156271

[18] Seufert, F., Chung, Y. K., Hildebrand, P. W. & Langenhan, T. 7TM domain structures of adhesion GPCRs: what’s new and what’s missing? *Trends Biochem. Sci.* **48**, 726–739 (2023). 37349240 10.1016/j.tibs.2023.05.007

[19] Mao, C. et al. Conformational transitions and activation of the adhesion receptor CD97. *Mol. Cell* **84**, 570–583 (2024). 10.1016/j.molcel.2023.12.020 38215752

[20] Scholz, N. et al. The adhesion GPCR latrophilin/CIRL shapes mechanosensation. *Cell Rep.* **11**, 866–874 (2015). 25937282 10.1016/j.celrep.2015.04.008

[21] Petersen, S. C. et al. The adhesion GPCR GPR126 has distinct, domain-dependent functions in schwann cell development mediated by interaction with Laminin-211. *Neuron* **85**, 755–769 (2015). 25695270 10.1016/j.neuron.2014.12.057 PMC4335265

[22] Wilde, C. et al. The constitutive activity of the adhesion GPCR GPR114/ADGRG5 is mediated by its tethered agonist. *FASEB J.* **30**, 666–673 (2016). 26499266 10.1096/fj.15-276220

[23] Liu, D. et al. CD97 promotes spleen dendritic cell homeostasis through the mechanosensing of red blood cells. *Science* **375**, eabi5965 (2022). 35143305 10.1126/science.abi5965 PMC9310086

[24] Boyden, S. E. et al. Vibratory Urticaria Associated with a Missense Variant in ADGRE2. *N. Engl. J. Med.* **374**, 656–663 (2016). 26841242 10.1056/NEJMoa1500611 PMC4782791

[25] Scholz, N. et al. Molecular sensing of mechano- and ligand-dependent adhesion GPCR dissociation. *Nature* **615**, 945–953 (2023). 36890234 10.1038/s41586-023-05802-5

[26] Fu, C. et al. Unveiling Mechanical Activation: GAIN Domain Unfolding and Dissociation in Adhesion GPCRs. Nano Lett. **23**, 9179–9186 (2023). 10.1021/acs.nanolett.3c01163 PMC10607210 37831892

[27] Dumas, L. et al. Uncovering and engineering the mechanical properties of the adhesion GPCR ADGRG1 GAIN domain. Preprint at 10.1101/2023.04.05.535724 (2023).

[28] Beliu, G. et al. Tethered agonist exposure in intact adhesion/class B2 GPCRs through intrinsic structural flexibility of the GAIN domain. *Mol. Cell* **81**, 905–921 (2021). 33497605 10.1016/j.molcel.2020.12.042

[29] Zhong, B. L. et al. Piconewton forces mediate GAIN domain dissociation of the Latrophilin-3 adhesion GPCR. *Nano Lett.* **23**, 9187–9194 (2023). 37831891 10.1021/acs.nanolett.3c03171 PMC11801148

[30] Xiao, P. et al. Tethered peptide activation mechanism of the adhesion GPCRs ADGRG2 and ADGRG4. *Nature* **604**, 771–778 (2022). 35418677 10.1038/s41586-022-04590-8

[31] Ping, Y. Q. et al. Structures of the glucocorticoid-bound adhesion receptor GPR97–Go complex. *Nature* **589**, 620–626 (2021). 33408414 10.1038/s41586-020-03083-w

[32] Barros-Álvarez, X. et al. The tethered peptide activation mechanism of adhesion GPCRs. *Nature* **604**, 757–762 (2022). 35418682 10.1038/s41586-022-04575-7 PMC9841879

[33] Ping, Y. Q. et al. Structural basis for the tethered peptide activation of adhesion GPCRs. *Nature* **604**, 763–770 (2022). 35418678 10.1038/s41586-022-04619-y

[34] Sun, Y. et al. Optimization of a peptide ligand for the adhesion GPCR ADGRG2 provides a potent tool to explore receptor biology. *J. Biol. Chem.* **296**, 100174 (2021). 33303626 10.1074/jbc.RA120.014726 PMC7948503

[35] Leon, K. et al. Structural basis for adhesion G protein-coupled receptor Gpr126 function. *Nat. Commun.* **11**, 194 (2020). 31924782 10.1038/s41467-019-14040-1 PMC6954182

[36] Salzman, G. S. et al. Structural basis for regulation of GPR56/ADGRG1 by its alternatively spliced extracellular domains. *Neuron* **91**, 1292–1304 (2016). 27657451 10.1016/j.neuron.2016.08.022 PMC5036532

[37] Chu, T. Y. et al. GPR97 triggers inflammatory processes in human neutrophils via a macromolecular complex upstream of PAR2 activation. *Nat. Commun.* **13**, 6385 (2022). 36302784 10.1038/s41467-022-34083-1 PMC9613636

[38] Ballesteros, J. A. & Weinstein, H. Integrated methods for the construction of three-dimensional models and computational probing of structure-function relations in G protein-coupled receptors. *Methods Neurosci.* **25**, 366–428 (1995).

[39] Isberg, V. et al. Generic GPCR residue numbers - Aligning topology maps while minding the gaps. *Trends Pharmacol. Sci.* **36**, 22–31 (2015). 25541108 10.1016/j.tips.2014.11.001 PMC4408928

[40] Wootten, D., Simms, J., Miller, L. J., Christopoulos, A. & Sexton, P. M. Polar transmembrane interactions drive formation of ligand-specific and signal pathway-biased family B G protein-coupled receptor conformations. *Proc. Natl Acad. Sci. USA* **110**, 5211–5216 (2013). 23479653 10.1073/pnas.1221585110 PMC3612682

[41] Pándy-Szekeres, G. et al. GPCRdb in 2023: state-specific structure models using AlphaFold2 and new ligand resources. *Nucleic Acids Res.* **51**, D395–D402 (2022). 10.1093/nar/gkac1013 PMC9825476 36395823

[42] Linden, O. P. J., van, Kooistra, A. J., Leurs, R., Esch, I. J. Pde & Graaf, C. de. KLIFS: A knowledge-Based structural database to navigate kinase–ligand interaction space. *J. Med. Chem.* **57**, 249–277 (2014). 23941661 10.1021/jm400378w

[43] Kanev, G. K., de Graaf, C., Westerman, B. A., de Esch, I. J. P. & Kooistra, A. J. KLIFS: an overhaul after the first 5 years of supporting kinase research. *Nucleic Acids Res.* **49**, gkaa895 (2020). 10.1093/nar/gkaa895 PMC7778968 33084889

[44] Kanev, G. K. et al. The landscape of atypical and eukaryotic protein kinases. *Trends Pharmacol. Sci.* **40**, 818–832 (2019). 31677919 10.1016/j.tips.2019.09.002

[45] Flock, T. et al. Universal allosteric mechanism for Gα activation by GPCRs. *Nature* **524**, 173–179 (2015). 26147082 10.1038/nature14663 PMC4866443

[46] Sente, A. et al. Molecular mechanism of modulating arrestin conformation by GPCR phosphorylation. *Nat. Struct. Mol. Biol.* **25**, 538–545 (2018). 29872229 10.1038/s41594-018-0071-3 PMC6101189

[47] Mirdita, M. et al. ColabFold: making protein folding accessible to all. *Nat. Methods* **19**, 679–682 (2022). 35637307 10.1038/s41592-022-01488-1 PMC9184281

[48] Jumper, J. et al. Highly accurate protein structure prediction with AlphaFold. *Nature* **596**, 583–589 (2021). 34265844 10.1038/s41586-021-03819-2 PMC8371605

[49] Herrera, L. P. T. et al. GPCRdb in 2025: adding odorant receptors, data mapper, structure similarity search and models of physiological ligand complexes. *Nucleic Acids Res.* gkae1065 10.1093/nar/gkae1065 (2024). 10.1093/nar/gkae1065 PMC11701689 39558158

[50] Kooistra, A. J. et al. GPCRdb in 2021: Integrating GPCR sequence, structure and function. *Nucleic Acids Res.* **49**, D335–D343 (2021). 33270898 10.1093/nar/gkaa1080 PMC7778909

[51] Pándy-Szekeres, G. et al. GPCRdb in 2018: Adding GPCR structure models and ligands. *Nucleic Acids Res.* **46**, D440–D446 (2018). 29155946 10.1093/nar/gkx1109 PMC5753179

[52] Krissinel, E. Enhanced fold recognition using efficient short fragment clustering. *J. Mol. Biochem.* **1**, 76 (2012). 27882309 PMC5117261

[53] Isberg, V. et al. GPCRDB: an information system for G protein-coupled receptors. *Nucleic Acids Res.* **42**, D422–D425 (2014). 24304901 10.1093/nar/gkt1255 PMC3965068

[54] Pérez-Hernández, G. et al. mdciao: Accessible analysis and visualization of molecular dynamics simulation data. Preprint at 10.1101/2022.07.15.500163 (2022). 10.1371/journal.pcbi.1012837 PMC12011235 40258031

[55] Moreno-Salinas, A. L. et al. Convergent selective signaling impairment exposes the pathogenicity of latrophilin-3 missense variants linked to inheritable ADHD susceptibility. *Mol. Psychiatry* **27**, 2425–2438 (2022). 35393556 10.1038/s41380-022-01537-3 PMC9135631

[56] Avila-Zozaya, M., Rodríguez-Hernández, B., Monterrubio-Ledezma, F., Cisneros, B. & Boucard, A. A. Thwarting of Lphn3 functions in cell motility and signaling by cancer-related GAIN domain somatic mutations. *Cells* **11**, 1913 (2022). 35741042 10.3390/cells11121913 PMC9221416

[57] Wright, S. C. et al. A conserved molecular switch in Class F receptors regulates receptor activation and pathway selection. *Nat. Commun.* **10**, 667 (2019). 10.1038/s41467-019-08630-2 PMC6368630 30737406

[58] Lin, H. et al. Structures of the ADGRG2–Gs complex in apo and ligand-bound forms. *Nat. Chem. Biol.* **18**, 1196–1203(2022). 10.1038/s41589-022-01084-6 35982227

[59] Bernadyn, T. F., Vizurraga, A., Adhikari, R., Kwarcinski, F. & Tall, G. G. GPR114/ADGRG5 is activated by its tethered-peptide-agonist because it is a cleaved adhesion GPCR. *J. Biol. Chem.* **299**, 105223 (2023). 10.1016/j.jbc.2023.105223 PMC10622838 37673336

[60] Kishore, A., Purcell, R. H., Nassiri-Toosi, Z. & Hall, R. A. Stalk-dependent and stalk-independent signaling by the adhesion G protein-coupled receptors GPR56 (ADGRG1) and BAI1 (ADGRB1). *J. Biol. Chem.* **291**, 3385–3394 (2016). 26710850 10.1074/jbc.M115.689349 PMC4751382

[61] Müller, A. et al. Oriented Cell Division in the C. elegans Embryo Is Coordinated by G-Protein Signaling Dependent on the Adhesion GPCR LAT-1. *PLoS Genet.* **11**, 10.1371/journal.pgen.1005624 (2015). 10.1371/journal.pgen.1005624 PMC4624771 26505631

[62] Scholz, N. et al. Mechano-dependent signaling by latrophilin/CIRL quenches cAMP in proprioceptive neurons. *ELife* **6**, 10.7554/elife.28360 (2017). 10.7554/eLife.28360 PMC5548486 28784204

[63] Monk, K. R. et al. A G Protein–coupled receptor is essential for schwann cells to initiate myelination. *Science* **325**, 1402–1405 (2009). 19745155 10.1126/science.1173474 PMC2856697

[64] Langenhan, T. et al. Model organisms in G protein–coupled receptor research. *Mol. Pharmacol.* **88**, 596–603 (2015). 25979002 10.1124/mol.115.098764 PMC4551050

[65] Bergmann, C. et al. Polycystic kidney disease. *Nat. Rev. Dis. Prim.* **4**, 50 (2018). 30523303 10.1038/s41572-018-0047-y PMC6592047

[66] Qian, F. et al. Cleavage of polycystin-1 requires the receptor for egg jelly domain and is disrupted by human autosomal-dominant polycystic kidney disease 1-associated mutations. *Proc. Natl. Acad. Sci. USA* **99**, 16981–16986 (2002). 12482949 10.1073/pnas.252484899 PMC139255

[67] Yu, S. et al. Essential role of cleavage of Polycystin-1 at G protein-coupled receptor proteolytic site for kidney tubular structure. *Proc. Natl. Acad. Sci. USA* **104**, 18688–18693 (2007). 18003909 10.1073/pnas.0708217104 PMC2141838

[68] Wei, W., Hackmann, K., Xu, H., Germino, G. & Qian, F. Characterization of cis-autoproteolysis of polycystin-1, the product of human polycystic kidney disease 1 gene. *J. Biol. Chem.* **282**, 21729–21737 (2007). 17525154 10.1074/jbc.M703218200

[69] Scholz, N., Langenhan, T. & Schöneberg, T. Revisiting the classification of adhesion GPCRs. *Ann. N. Y. Acad. Sci.* **1456**, 80–95 (2019). 31365134 10.1111/nyas.14192 PMC6900090

[70] Nordström, K. J. V., Lagerström, M. C., Wallér, L. M. J., Fredriksson, R. & Schiöth, H. B. The secretin GPCRs descended from the family of adhesion GPCRs. *Mol. Biol. Evol.* **26**, 71–84 (2009). 18845549 10.1093/molbev/msn228

[71] Dohrmann, M. & Wörheide, G. Dating early animal evolution using phylogenomic data. *Sci. Rep.* **7**, 3599 (2017). 28620233 10.1038/s41598-017-03791-w PMC5472626

[72] Krishnan, A. et al. The GPCR repertoire in the demosponge Amphimedon queenslandica: insights into the GPCR system at the early divergence of animals. *BMC Evol. Biol.* **14**, 270 (2014). 25528161 10.1186/s12862-014-0270-4 PMC4302439

[73] Illergård, K., Ardell, D. H. & Elofsson, A. Structure is three to ten times more conserved than sequence—A study of structural response in protein cores. *Proteins Struct. Funct. Bioinform.* **77**, 499–508 (2009). 10.1002/prot.22458 19507241

[74] Piao, X. et al. Genotype–phenotype analysis of human frontoparietal polymicrogyria syndromes. *Ann. Neurol.* **58**, 680–687 (2005). 16240336 10.1002/ana.20616

[75] Chang, G.-W. et al. The adhesion G protein-coupled receptor GPR56/ADGRG1 is an inhibitory receptor on human NK cells. *Cell Rep.* **15**, 1757–1770 (2016). 27184850 10.1016/j.celrep.2016.04.053

[76] Russell, R. B. & Barton, G. J. Multiple protein sequence alignment from tertiary structure comparison: Assignment of global and residue confidence levels. *Proteins Struct. Funct. Bioinform.* **14**, 309–323 (1992). 10.1002/prot.340140216 1409577

[77] Frishman, D. & Argos, P. Knowledge‐based protein secondary structure assignment. *Proteins Struct. Funct. Bioinform.* **23**, 566–579 (1995). 10.1002/prot.340230412 8749853

[78] Agirre, J. et al. The CCP4 suite: integrative software for macromolecular crystallography. *Acta Crystallogr. Sect. D Struct. Biol.* **79**, 449–461 (2023). 37259835 10.1107/S2059798323003595 PMC10233625

[79] Pedregosa, F. et al. Scikit-learn: Machine learning. *Python. J. Mach. Learn. Res.* **12**, 2825–2830 (2011).

[80] Varadi, M. et al. AlphaFold protein structure database: massively expanding the structural coverage of protein-sequence space with high-accuracy models. *Nucleic Acids Res.* **50**, D439–D444 (2022). 34791371 10.1093/nar/gkab1061 PMC8728224

[81] Ng, P. C. & Henikoff, S. Predicting deleterious amino acid substitutions. *Genome Res.* **11**, 863–874 (2001). 11337480 10.1101/gr.176601 PMC311071

[82] Adzhubei, I., Jordan, D. M. & Sunyaev, S. R. Predicting functional effect of human missense mutations using PolyPhen-2. *Curr. Protoc. Hum. Genet.*10.1002/0471142905.hg0720s76 (2013). 10.1002/0471142905.hg0720s76 PMC4480630 23315928

[83] Seufert F. et al. Generic residue numbering of the GAIN domain of adhesion GPCRs. Generic residue numbering of the GAIN domain of adhesion GPCRs. 10.5281/zenodo.12515544 (2024).

[84] Seufert F. et al. Generic residue numbering of the GAIN domain of adhesion GPCRs. FloSeu/GAIN-GRN: GAIN-GRN version 1.0. 10.5281/zenodo.14140353 (2024). 10.1038/s41467-024-55466-6 PMC11697300 39747076

[85] Munk, C., Harpsøe, K., Hauser, A. S., Isberg, V. & Gloriam, D. E. Integrating structural and mutagenesis data to elucidate GPCR ligand binding. *Curr. Opin. Pharmacol.* **30**, 51–58 (2016). 27475047 10.1016/j.coph.2016.07.003 PMC6910865

[86] Collins, R. L. et al. A structural variation reference for medical and population genetics. *Nature* **581**, 444–451 (2020). 32461652 10.1038/s41586-020-2287-8 PMC7334194

[87] Vincent, F. et al. Toward a shared vision for cancer genomic data. *N. Engl. J. Med.* **375**, 1109–1112 (2016). 27653561 10.1056/NEJMp1607591 PMC6309165

