# Supplementary material for: Generic residue numbering of the GAIN domain of adhesion GPCRs
Source: Nat Commun. 2025 Jan 2;16:246. doi: 10.1038/s41467-024-55466-6 (PMC11697300; doi:10.1038/s41467-024-55466-6)
Supplement: Supplementary file 1 — Supplementary Information [file 41467_2024_55466_MOESM1_ESM.pdf]

Generic residue numbering of the GAIN domain of adhesion GPCRs

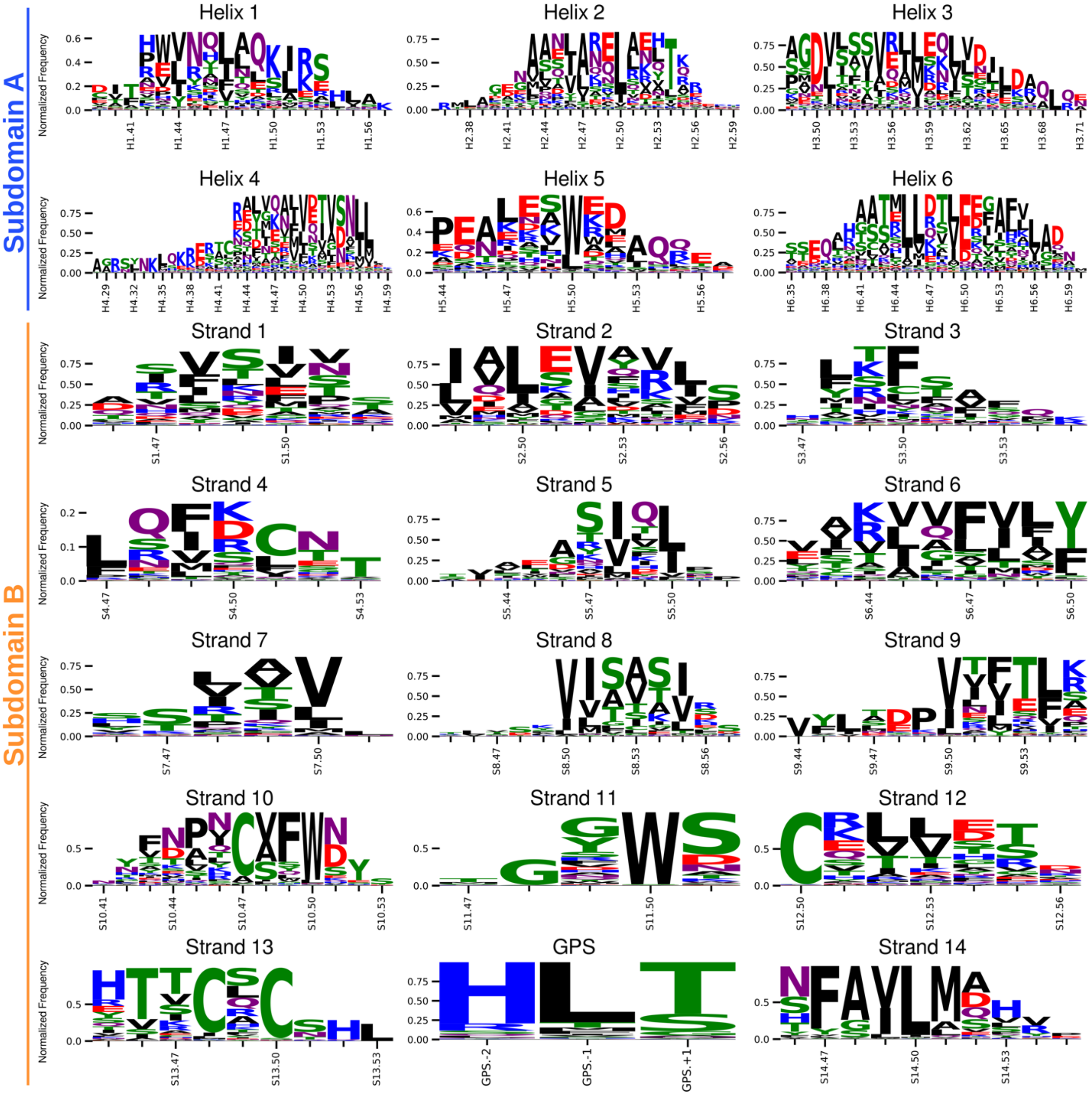

Supplementary Fig. 1 | Consensus Logo for all GAIN-GRN indexed segments. Residues are colored by chemical property. Total column height represents the total number of structures. The labeled position and letter height represents the relative fraction of residue present at the respective position, with a value of 1.0 indicating the GRN present in all 14,435 studied adhesion GPCR GAIN domains. Source data are provided as a Source Data file.

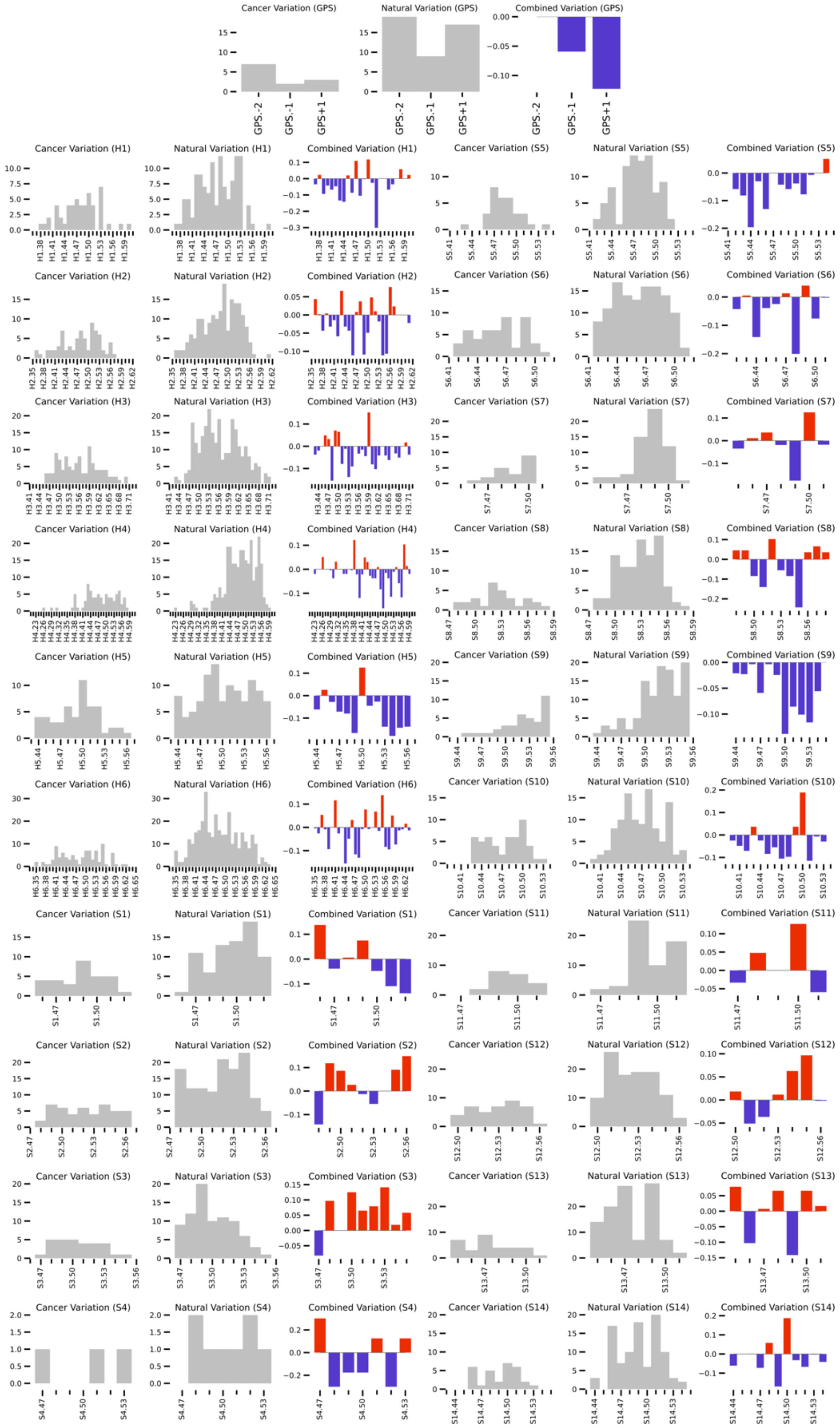
Cancer Variation (GPS)
Natural Variation (GPS)
Combined Variation (GPS)
Cancer Variation (H1)
Natural Variation (H1)
Combined Variation (H1)
Cancer Variation (S5)
Natural Variation (S5)
Combined Variation (S5)
Cancer Variation (H2)
Natural Variation (H2)
Combined Variation (H2)
Cancer Variation (S6)
Natural Variation (S6)
Combined Variation (S6)
Cancer Variation (H3)
Natural Variation (H3)
Combined Variation (H3)
Cancer Variation (S7)
Natural Variation (S7)
Combined Variation (S7)
Cancer Variation (H4)
Natural Variation (H4)
Combined Variation (H4)
Cancer Variation (S8)
Natural Variation (S8)
Combined Variation (S8)
Cancer Variation (H5)
Natural Variation (H5)
Combined Variation (H5)
Cancer Variation (S9)
Natural Variation (S9)
Combined Variation (S9)
Cancer Variation (H6)
Natural Variation (H6)
Combined Variation (H6)
Cancer Variation (S10)
Natural Variation (S10)
Combined Variation (S10)
Cancer Variation (S1)
Natural Variation (S1)
Combined Variation (S1)
Cancer Variation (S11)
Natural Variation (S11)
Combined Variation (S11)
Cancer Variation (S2)
Natural Variation (S2)
Combined Variation (S2)
Cancer Variation (S12)
Natural Variation (S12)
Combined Variation (S12)
Cancer Variation (S3)
Natural Variation (S3)
Combined Variation (S3)
Cancer Variation (S13)
Natural Variation (S13)
Combined Variation (S13)
Cancer Variation (S4)
Natural Variation (S4)
Combined Variation (S4)
Cancer Variation (S14)
Natural Variation (S14)
Combined Variation (S14)

Supplementary Fig. 2 | Cancer Enrichment Score for GAIN-GRN indexed Elements. Grey plots show number of cancer-associated variants from The Cancer Genome Atlas (TCGA) Genomic Data Commons (GDC)[1] and naturally occurring polymorphisms from the dbSNP. Right plots show cancer enrichment score according to Wright et al., 2019[2]. Source data are provided as a Source Data file.

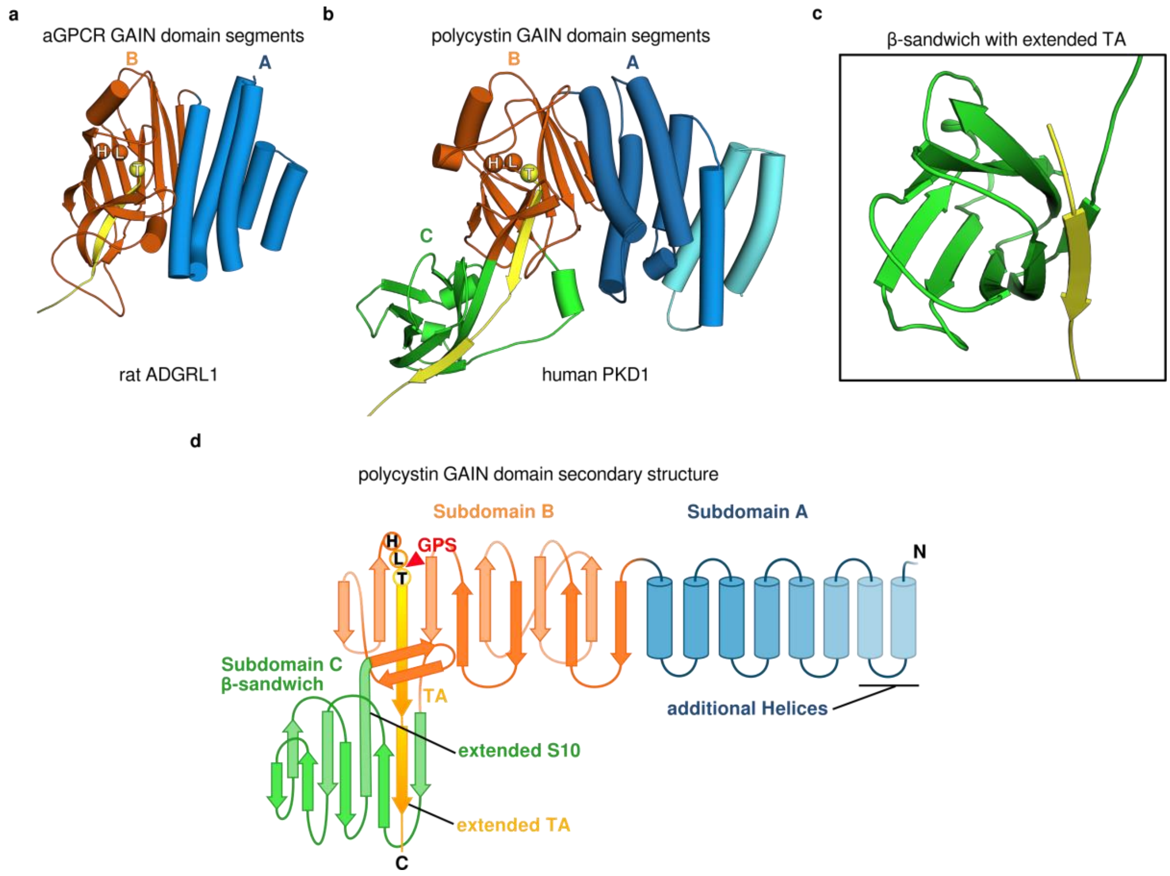

Supplementary Fig. 3 | The topology of polycystin GAIN domains. a, the adhesion GPCR GAIN domain shows up to six helices in subdomain A (blue) and up to 14 β-strands in a β-sandwich subdomain B (orange), with the tethered agonist (TA, yellow), forming the most C-terminal β-strand. The GPCR proteolysis site is denoted by the catalytic triad HL|T (labeled spheres) for autoproteolysis. **b**, the polycystin GAIN domain shows more helices in subdomain A (light blue for additional helices, blue), with identical topology of subdomain B and an additional subdomain C (green) forming a β-sandwich of nine strands with an extension of the TA embedded as its most C-terminal strand (**c**). **d**, the topology of the polycystin GAIN domain shows the additional helices and subdomain C as an extended fold between S9 and S10, where S10 may extend in β-strand conformation uninterrupted as part of both β-sandwich subdomains. Topology is based on human polycystin-1 (PKD1) protein (UniProt identifier: P98161). Figure created with PyMOL.

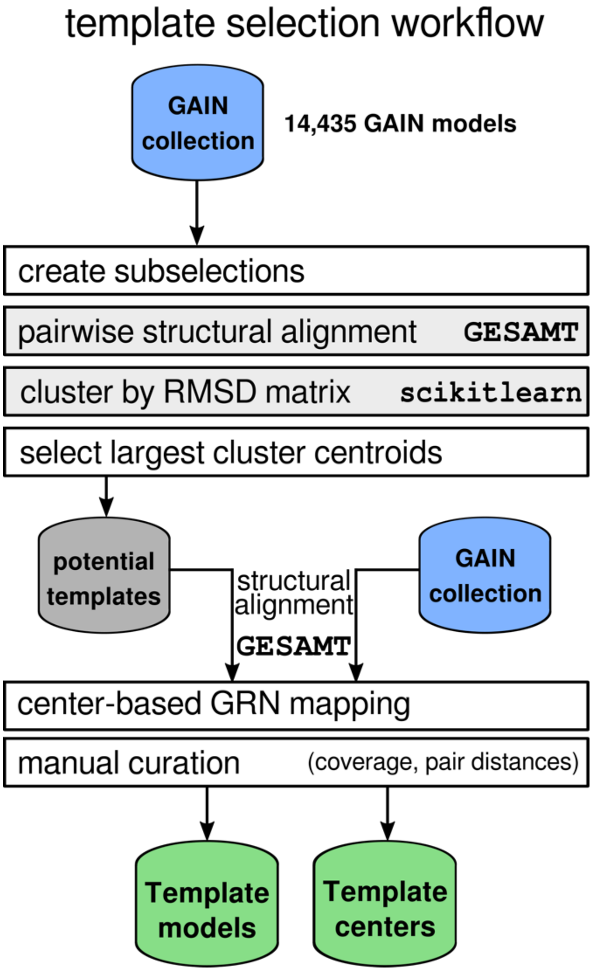

Supplementary Fig. 4 | Methodological workflow for template selection. The workflow proceeds top to bottom. Based on the dataset of 14,435 adhesion GPCR GAIN domain models (blue), sub-selections were created based on receptor orthologues, which were put through GESAMT pairwise alignment[10], to find cluster within the sub-selections with the scikitlearn[11] package. Of these clusters, the lowest-RMSD structure was added to the potential templates. Each of the potential template was pairwise aligned via GESAMT[10] and via manual curation, a set of template models covering all receptor orthologues was created and center positions (represented by the ".50" GRN) defined as the basis for the GAIN GRN calculation (green).

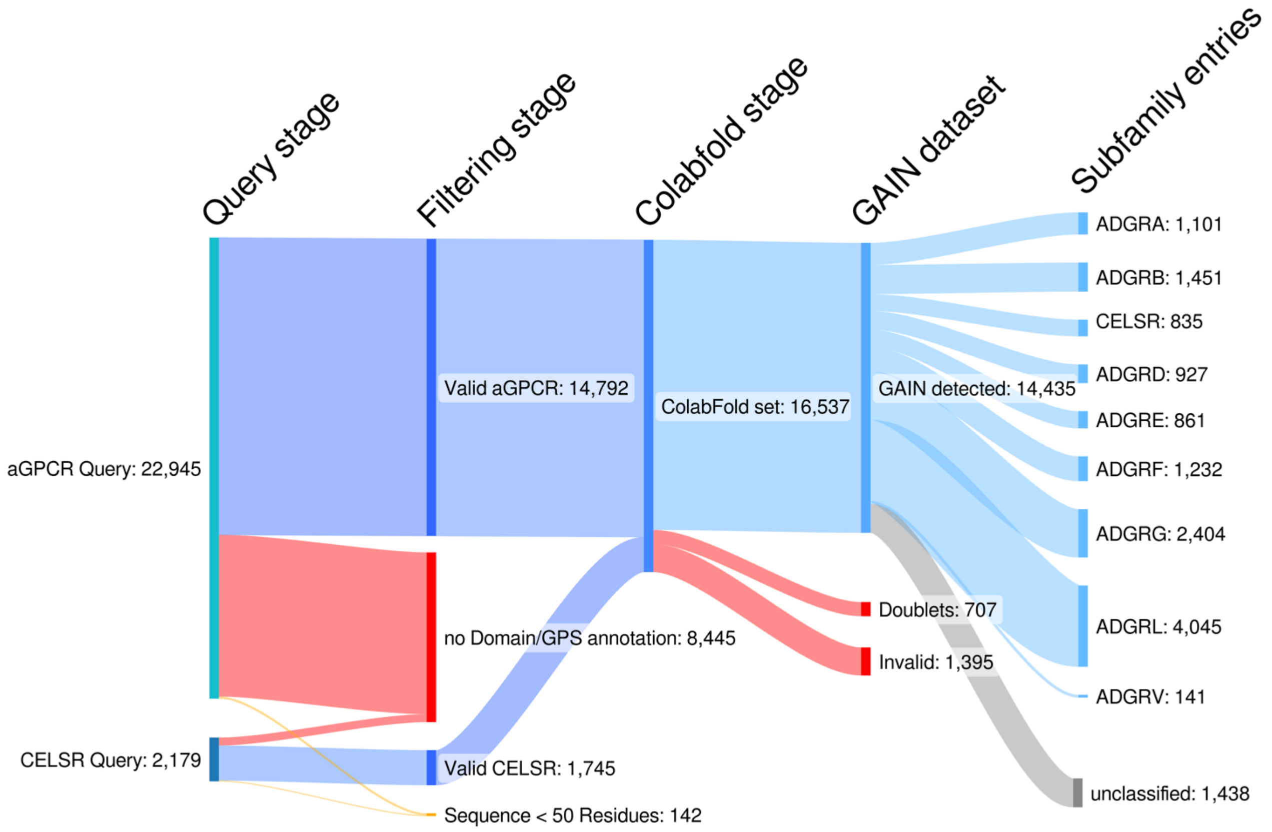

Supplementary Fig. 5 | Filtering and Composition of the aGPCR GAIN domain dataset for the GAIN GRN. From left to right: At the query stage, the sequences were retrieved in two queries from the UniProtKB database[12]. Entries were filtered for the "GPS" domain entry and a minimum sequence length of 50 residues. The remaining sequences were folded via ColabFold/AlphaFold2[13,14] and checked for valid GAIN domain architecture. The dataset decomposed by aGPCR subfamily is shown in the right.
Created with SankeyMATIC (https://sankeymatic.com).

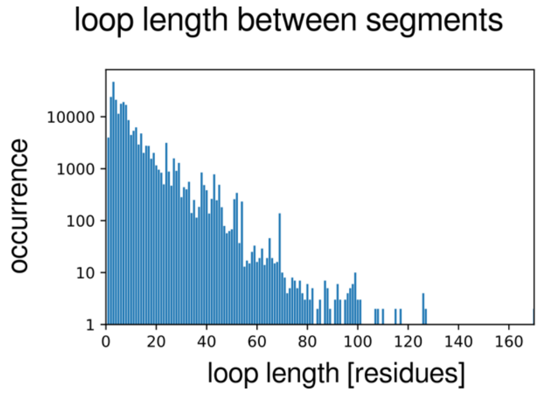

Supplementary Fig. 6 | Distribution of inter-segment loop lengths in the GAIN domain model dataset. Number of occurring loop with defined length is shown with logarithmic scale.

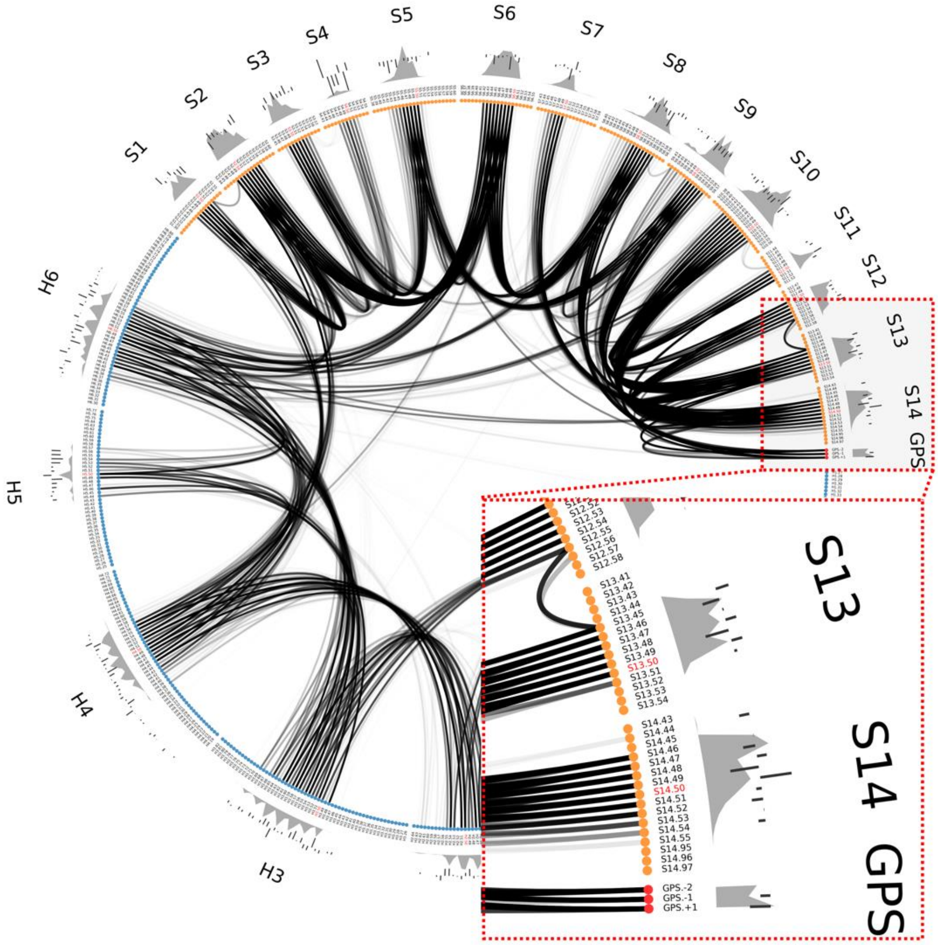

Supplementary Fig. 7 | aGPCR *contactome* visualized as a flareplot. Residues are represented as color-coded dots in a circle (blue and orange for subdomains A and B, respectively and red for GPS) grouped by their secondary structure elements (SSE). Pairs of dots are connected with black curves, with opacities proportional to each pair's residue-residue contact frequencies (4 Å distance cutoff) over the whole dataset of geometries (N= 14435, see Supplementary Fig. 5). Aggregated by residue, these frequencies yield a measure of residue participation in the overall GAIN-*contactome*. This measure is represented in gray on the outer ring of the plot (gray, ragged backdrop or *aura*). Also on that outer ring is the cancer enrichment score (Supplementary Fig. 2), which is represented as straight lines pointing either inwards (negative score) or outwards (positive score). Taking the shown inset as example, the following information can be extracted: of S14, residue S14.47 is the highest participating residue (highest gray *aura)* while not particularly cancer enriched (short straight line going inwards). The segment center, S14.50 (GRN label in red) is cancer enriched (longer straight line pointing outwards) and also one of the highest participating residues. For clarity, intra-segment contacts are not shown.

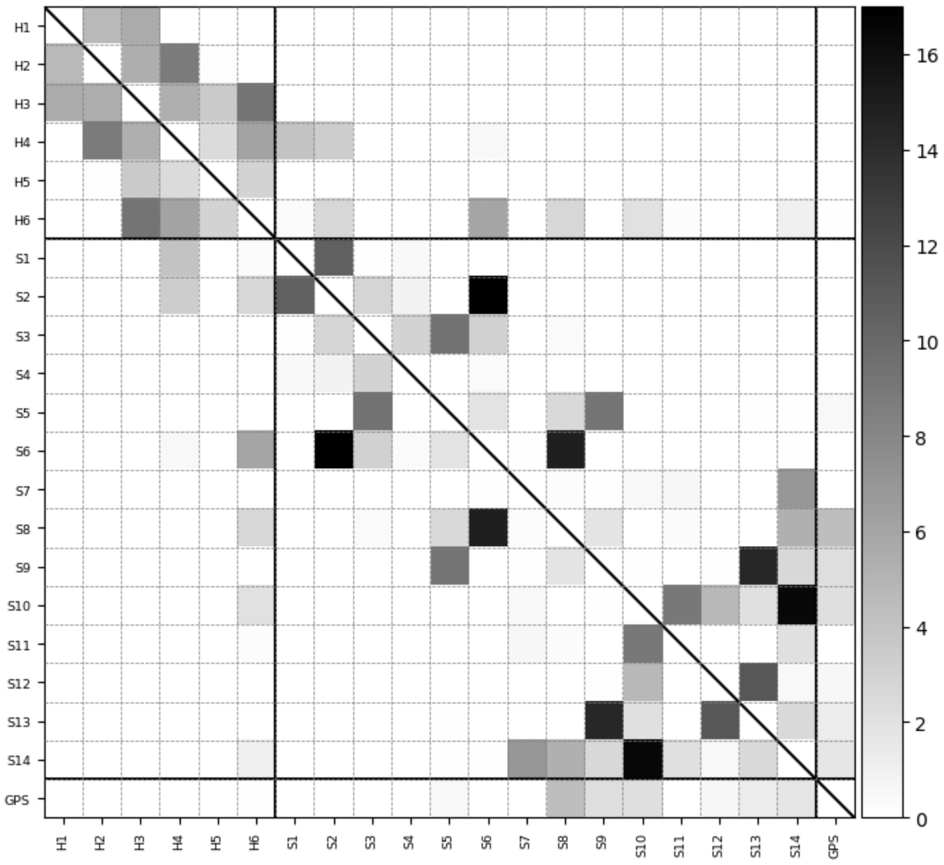

Supplementary Fig. 8 | Per-segment contact-map for intra- and inter-domain contacts. Average number of residue-residue contacts (4 Å distance cutoff) aggregated to each residue's GAIN segment. For clarity of the colormap, the diagonal has been left blank. The solid vertical and horizontal lines visually separate subdomains A, B and the GPS triad. Of note, H4 and H6 of subdomain A account for all inter-domain contacts (off-diagonal blocks in the upper right, or in the bottom left), contacting mostly S6 of subdomain B. This is in line with the observation about conserved SSEs of Figure 2b. For individual contacts between residues, see Supplementary Fig. 7.

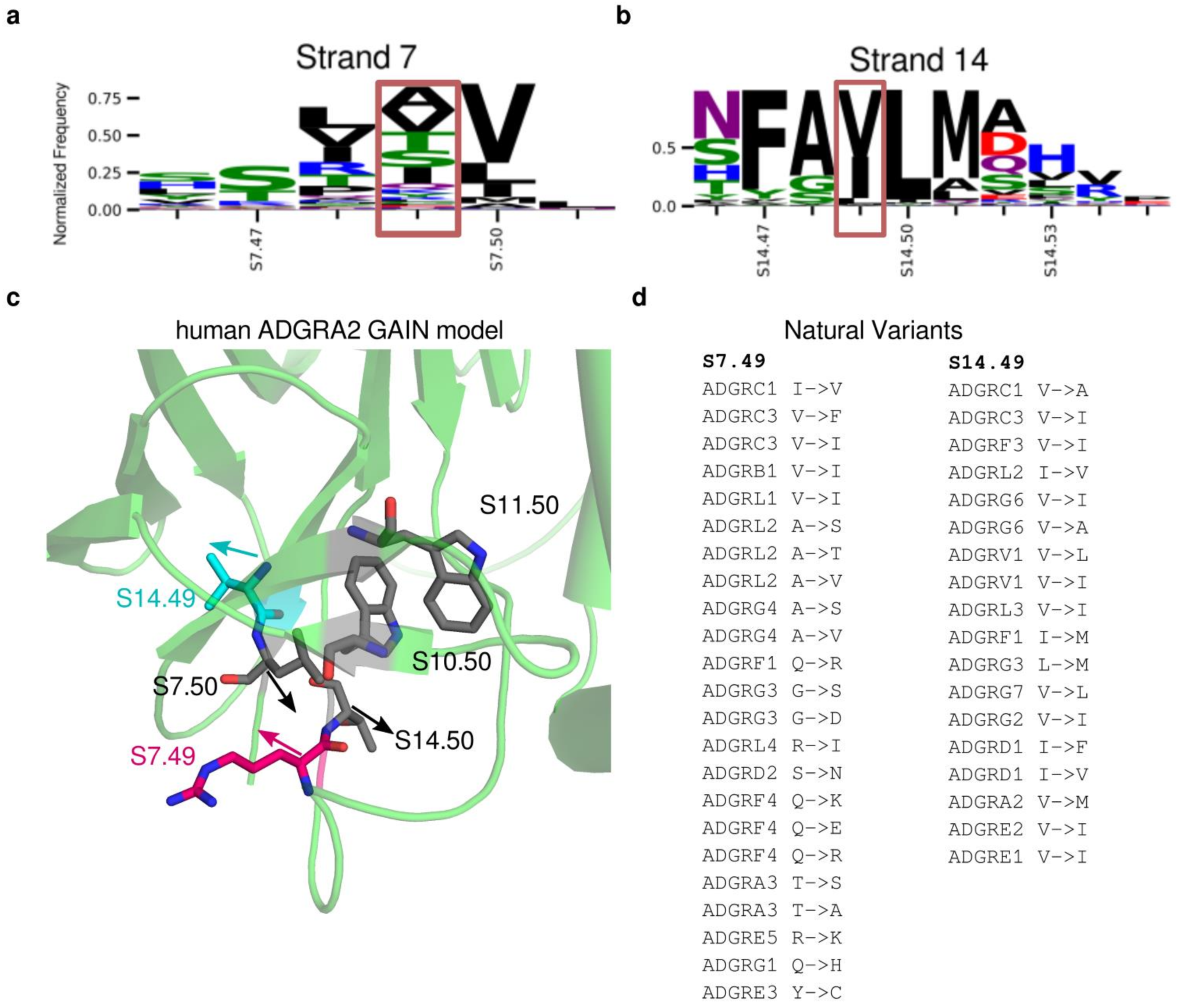

Supplementary Fig. 9 | Variant highlights of GRN labels S7.49 and S14.49. a, Logoplot of Strand 7 highlighting the high sequence variability at S7.49 with mainly small medium polar or non-aromatic residues A, V, T, S and I; **b**, Logoplot of Strand 14 shows mainly V, I and L at position S14.49; **c**, GAIN domain model of ADGRA2 with the “VWWL” motif indicated as grey sticks, VS14.49 in cyan and RS7.49 in hotpink. Cα-Cβ bond directions are indicated with colored arrows; **d**, consequence of natural variants in S7.49 and S14.49 show similar characteristics of residue substitutions. Figure created with PyMOL.

**Organisms with an ADGRA1 GAIN domain**	**Organisms with an ADGRE4 GAIN domain**
Amphiprion ocellaris	Acinonyx jubatus
Amphiprion percula	Ailuropoda melanoleuca
Anabas testudineus	Aotus nancymaae
Aquila chrysaetos chrysaetos.	Betta splendens
Astatotilapia calliptera	Bos indicus
Athene cunicularia	Bos indicus x Bos taurus
Aythya fuligula	Callorhinus ursinus
Betta splendens	Camelus dromedarius
Chanos chanos	Capra hircus
Columba livia	Carassius auratus
Cottoperca gobio	Enhydra lutris kenyoni
Crocodylus porosus	Equus caballus
Cynoglossus semilaevis	Felis catus
Esox lucius	Ictidomys tridecemlineatus
Ficedula albicollis	Larimichthys crocea
Geospiza fortis	Leptonychotes weddellii
Geotrypetes seraphini	Lynx canadensis
Gopherus agassizii	Mesocricetus auratus
Hucho hucho	Mus caroli
Ictalurus punctatus	Mus musculus
Labrus bergylta	Neomonachus schauinslandi
Lepidothrix coronata	Odocoileus virginianus texanus
Lepisosteus oculatus	Oryzias melastigma
Mastacembelus armatus	Ovis aries
Maylandia zebra	Pan paniscus
Meleagris gallopavo	Panthera pardus
Microcaecilia unicolor.	Paramormyrops kingsleyae
Mola mola	Peromyscus maniculatus bairdii
Monopterus albus	Phyllostomus discolor
Myripristis murdjan	Pteropus vampyrus
Notechis scutatus	Puma concolor
Oryzias melastigma	Rattus norvegicus
Pantherophis guttatus	Sapajus apella
Parambassis ranga	Sarcophilus harrisii
Paramormyrops kingsleyae.	Trichechus manatus latirostris
Patagioenas fasciata monilis.	Ursus arctos horribilis
Phascolarctos cinereus	Vicugna pacos
Phasianus colchicus	Vulpes vulpes
Poecilia reticulata	Zalophus californianus
Pogona vitticeps	
Pygocentrus nattereri	
Salarias fasciatus	
Sarcophilus harrisii	
Seriola dumerili	
Sparus aurata	
Takifugu rubripes	
Tetraodon nigroviridis	

Supplementary Table 1 | List of organisms with ADGRA1 and ADGRE4 that contain a GAIN domain.

GRN contact	counts	frequency
**H6.53-S6.46**	11965	0.83
**H4.50-S1.50**	10695	0.74
**H4.50-S2.48**	9953	0.69
**H6.43-S10.50**	9329	0.65
**H6.53-S2.52**	8713	0.60
**H6.54-S8.54**	8002	0.55
**H6.46-S14.46**	7210	0.50
**H4.57-S6.50**	4207	0.29
**H6.56-S1.48**	2171	0.15
**H6.39-S11.48**	1236	0.09
**H3.66-S8.56**	293	0.02
**H3.55-S2.48**	111	0.01
**H3.52-S1.50**	111	0.01
**H4.50-S10.50**	84	0.01
**H5.47-S7.49**	50	0.00
**H4.54-S8.54**	46	0.00
**H3.62-S6.50**	40	0.00
**H4.50-S14.46**	25	0.00
**H5.44-S2.48**	16	0.00
**H5.47-S6.50**	15	0.00

Supplementary Table 2 | Most frequent (N=14435) inter-domain, inter segment consensus contacts, excluding those of the GPS triad. The segment centers have been highlighted in red, as in Supplementary Fig. 7. For the contacts of the GPS triad, see Ext. Data. Table 2. Covalently bound residues are excluded.

GRN contacts	N	frequency
**GPS.-2-S13.50**	14410	1.00
**GPS.+1-S14.47**	14309	0.99
**GPS.+1-S8.53**	14160	0.98
**GPS.+1-S10.49**	11830	0.82
**GPS.-2-S9.50**	9817	0.68
**GPS.-2-S12.50**	7661	0.53
**GPS.-1-S5.50**	6639	0.46
**GPS.+1-H6.50**	1156	0.08
**GPS.+1-S7.56**	81	0.01
**GPS.-2-S11.51**	46	0.00

Supplementary Table 3 | Most frequent (N=14435) consensus contacts for the GPS triad for each segment. The segments where the GPS triad is embedded (cleaved or uncleaved) appear as the most frequent (S13, S14) interaction partners. Notably, even H6 of subdomain a A is in contact with the GPS sometimes. Covalently bound residues are excluded. Segment centers have been highlighted in red, as in Supplementary Fig. 7.

1. Araç, D. *et al.* A novel evolutionarily conserved domain of cell-adhesion GPCRs mexadiates autoproteolysis. *The EMBO Journal* **31**, 1364–1378 (2012).

2. Munk, C., Harpsøe, K., Hauser, A. S., Isberg, V. & Gloriam, D. E. Integrating structural and mutagenesis data to elucidate GPCR ligand binding. *Curr. Opin. Pharmacol.* **30**, 51–58 (2016).

3. Isberg, V. *et al.* Generic GPCR residue numbers - Aligning topology maps while minding the gaps. *Trends in Pharmacological Sciences* **36**, 22–31 (2015).

4. Isberg, V. *et al.* GPCRDB: an information system for G protein-coupled receptors. *Nucleic Acids Res.* **42**, D422–D425 (2014).

5. Kooistra, A. J. *et al.* GPCRdb in 2021: Integrating GPCR sequence, structure and function. *Nucleic Acids Research* **49**, D335–D343 (2021).

6. Linden, O. P. J. van, Kooistra, A. J., Leurs, R., Esch, I. J. P. de & Graaf, C. de. KLIFS: A Knowledge-Based Structural Database To Navigate Kinase–Ligand Interaction Space. *J. Med. Chem.* **57**, 249–277 (2014).

7. Collins, R. L. *et al.* A structural variation reference for medical and population genetics. *Nature* **581**, 444–451 (2020).

8. Vincent, F. *et al.* Toward a Shared Vision for Cancer Genomic Data. *N. Engl. J. Med.* **375**, 1109–1112 (2016).

9. Wright, S. C. *et al.* A conserved molecular switch in Class F receptors regulates receptor activation and pathway selection. *Nature Communications* **10**, (2019).

10. Krissinel, E. Enhanced fold recognition using efficient short fragment clustering. *Journal of molecular biochemistry* **1**, 76 (2012).

11. Pedregosa, F. *et al.* Scikit-Learn: Machine Learning in Python. *J. Mach. Learn. Res.* **12**, 2825–2830 (2011).

12. Consortium, T. U. *et al.* UniProt: the Universal Protein Knowledgebase in 2023. *Nucleic Acids Res.* **51**, D523–D531 (2023).

13. Jumper, J. *et al.* Highly accurate protein structure prediction with AlphaFold. *Nature* **596**, 583–589 (2021).

14. Mirdita, M. *et al.* ColabFold: making protein folding accessible to all. *Nature Methods* **19**, 679–682 (2022).
