# Supplementary material for: Generic residue numbering of the GAIN domain of adhesion GPCRs
Source: Nat Commun. 2025 Jan 2;16:246. doi: 10.1038/s41467-024-55466-6 (PMC11697300; doi:10.1038/s41467-024-55466-6)
Supplement: Supplementary file 2 — Description of Additional Supplementary Files [file 41467_2024_55466_MOESM2_ESM.pdf]

Description of Additional Supplementary Files

File name: Supplementary Data 1
Description: Full overview of contact frequencies between GAIN-GRN indexed residues. Frequencies correspond to the fraction of GAIN domain models containing respective contact relative to the total GAIN domain dataset.
